# Comprehensive Analyses of Ventricular Myocyte Models Identify Targets Exhibiting Favorable Rate Dependence

**DOI:** 10.1371/journal.pcbi.1003543

**Published:** 2014-03-27

**Authors:** Megan A. Cummins, Pavan J. Dalal, Marco Bugana, Stefano Severi, Eric A. Sobie

**Affiliations:** 1 Department of Pharmacology and Systems Therapeutics, Icahn School of Medicine at Mount Sinai, New York, New York, United States of America; 2 University of Bologna, Bologna, Italy; University of California San Diego, United States of America

## Abstract

Reverse rate dependence is a problematic property of antiarrhythmic drugs that prolong the cardiac action potential (AP). The prolongation caused by reverse rate dependent agents is greater at slow heart rates, resulting in both reduced arrhythmia suppression at fast rates and increased arrhythmia risk at slow rates. The opposite property, forward rate dependence, would theoretically overcome these parallel problems, yet forward rate dependent (FRD) antiarrhythmics remain elusive. Moreover, there is evidence that reverse rate dependence is an intrinsic property of perturbations to the AP. We have addressed the possibility of forward rate dependence by performing a comprehensive analysis of 13 ventricular myocyte models. By simulating populations of myocytes with varying properties and analyzing population results statistically, we simultaneously predicted the rate-dependent effects of changes in multiple model parameters. An average of 40 parameters were tested in each model, and effects on AP duration were assessed at slow (0.2 Hz) and fast (2 Hz) rates. The analysis identified a variety of FRD ionic current perturbations and generated specific predictions regarding their mechanisms. For instance, an increase in L-type calcium current is FRD when this is accompanied by indirect, rate-dependent changes in slow delayed rectifier potassium current. A comparison of predictions across models identified inward rectifier potassium current and the sodium-potassium pump as the two targets most likely to produce FRD AP prolongation. Finally, a statistical analysis of results from the 13 models demonstrated that models displaying minimal rate-dependent changes in AP shape have little capacity for FRD perturbations, whereas models with large shape changes have considerable FRD potential. This can explain differences between species and between ventricular cell types. Overall, this study provides new insights, both specific and general, into the determinants of AP duration rate dependence, and illustrates a strategy for the design of potentially beneficial antiarrhythmic drugs.

## Author Summary

Several drugs intended to treat cardiac arrhythmias have failed because of unfavorable rate-dependent properties. That is, the drugs fail to alter electrical activity at fast heart rates, where this would be beneficial, but they do affect electrical activity at slow rates, where this is unwanted. In targeted studies, several agents have been shown to exhibit these unfavorable properties, suggesting that these rate-dependent responses may be intrinsic to ventricular muscle. To determine whether drugs with desirable rate-dependent properties could be rationally designed, we performed comprehensive and systematic analyses of several heart cell models. These analyses calculated the rate-dependent properties of changes in any model parameter, thereby generating simultaneously a large number of model predictions. The analyses showed that targets with favorable rate-dependent properties could indeed be identified, and further simulations uncovered the mechanisms underlying these behaviors. Moreover, a quantitative comparison of results obtained in different models provided new insight in why a given drug applied to different species, or to different tissue types, might produce different rate-dependent behaviors. Overall this study shows how a comprehensive and systematic approach to heart cell models can both identify novel targets and produce more general insight into rate-dependent alterations to cardiac electrical activity.

## Introduction

Prolongation of the ventricular action potential (AP), the action of class III antiarrhythmic drugs, is an antiarrhythmic strategy with a checkered past. Theoretically, increasing the action potential duration (APD) should be antiarrhythmic; a longer APD will increase the refractory period of the myocardium, thereby inhibiting the pathological re-entry of excitation that underlies many arrhythmias. However, a large-scale clinical trial that tested the Class III antiarrhythmic d-sotalol demonstrated increased rather than decreased mortality with this agent, presumably due to increased ventricular arrhythmias [1]. Moreover, multiple lines of evidence implicate a long QT interval, the electrocardiographic marker for prolonged APD, in arrhythmogenesis, including the increased incidence of torsades de pointes (TdP) and sudden cardiac death in both congenital and acquired long QT syndromes [2]. Indeed, safety pharmacology screens routinely test for QT prolongation as an unwanted side effect of novel candidate pharmaceutics [3]. How can AP prolongation simultaneously be both antiarrhythmic in principle and an indicator of proarrhythmia in practice? More nuanced analyses have suggested that QT and AP prolongation are not *per se* arrhythmogenic, but become so when accompanied by reverse rate dependence [4].

Reverse rate dependent (RRD) action potential prolongation refers to the phenomenon that drugs will prolong the APD to a greater extent at slow heart rates than at fast [5]. RRD prolongation is an unfortunate yet common property of Class III antiarrhythmics. This is doubly problematic, as it not only weakens the ability of these drugs to suppress re-entrant tachyarrhythmias, but also, because of the exaggerated action at slow heart rates, increases *pro*arrhythmic potential. The excessive increase in APD at slow heart rates, when the APD is naturally longer to begin with, allows for recovery of inactivated calcium channels, increasing the likelihood of an early afterdepolarization [6], a cellular event that can precipitate TdP [7]–[8].

This lack of efficacy at rapid rates and arrhythmogenic potential at slow rates makes reverse rate dependence undesirable and reduces the therapeutic potential of class III agents. An ideal class III agent would instead prolong APs in a *forward* rate dependent (FRD) manner. That is, it would prolong the APD at fast heart rates but induce minimal prolongation at slow heart rates [5]. Current class III agents prolong the APD primarily through inhibition of the rapid delayed rectifier K^+^ current (I_Kr_), and I_Kr_ block specifically has been shown to be RRD [9]. Since inhibition of any repolarizing current, or accentuation of any depolarizing current, could theoretically prolong the APD and increase the refractoriness of the myocardium, targets other than I_Kr_ have been under investigation [10]. Yet FRD agents have remained elusive. In fact, not only have many drugs been shown to exhibit reverse rate dependence, there is evidence that RRD behavior is not unique to I_Kr_-block, but instead occurs with multiple perturbations that affect the APD, including injection of membrane current [11]–[13].

Zaza has provided an elegant explanation, based on the rate of change of the membrane potential [14], for why multiple pharmacological agents exhibit RRD behavior. This idea is based on the observation that APD will be naturally shorter at faster than at slower heart rates in most mammals, including human, canine, and guinea pig. Because of the shortened APD, the rate of repolarization, dV/dt, will be larger in magnitude at faster rates. Zaza describes how a given change in membrane current that prolongs APD will inherently have a smaller effect when working against a larger rate of repolarization. Thus, at faster heart rates, any AP-prolonging perturbation must act against a stronger repolarizing force, and this attenuates the resulting prolongation, resulting in reverse rate dependence [14]. This explanation, combined with the RRD behavior seen in response to a variety of agents, has led to the idea that this is an inherent property of ventricular preparations [13].

The implication that reverse rate dependence is an inherent property of myocytes leads to the question of whether the search for an effective class III agent is hopeless. In other words, are all antiarrhythmic strategies based on prolonging APD destined to fail due to reverse rate dependence? In the present study, we have addressed this question by performing a comprehensive analysis of ventricular myocyte models. By simulating heterogeneous populations of ventricular myocytes and analyzing population results statistically [15], we simultaneously predicted the rate-dependent effects of changes to all model parameters. Our results show that, while ventricular cell models often display reverse rate dependence, a variety of ionic current perturbations do in fact produce the desired forward rate dependence. The perturbations we identify provide potential drug targets for FRD APD prolongation, and further simulations of these perturbations provide insight into how FRD behavior can be produced. Additionally, a comparison between mathematical models demonstrates that rate-dependent changes in AP shape play a central role in determining a myocyte's capacity for forward *versus* reverse rate dependence. Through these simulation results, this study provides new insight into the determinants of APD rate dependence and illustrates a strategy for the design of potentially beneficial antiarrhythmic drugs.

## Results

### Ventricular cell models can exhibit reverse rate dependence

**Figure 1:**
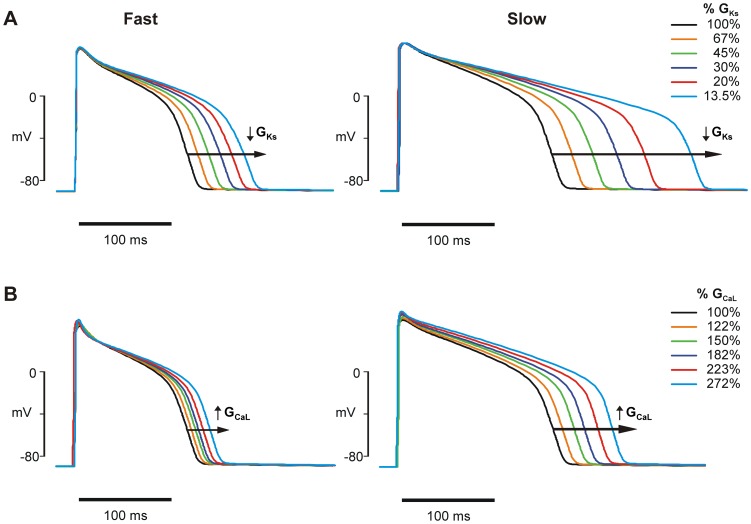
Reverse rate dependence of action potential prolongation. Reverse rate dependence is observed with perturbations to ionic current properties in a ventricular cell model, LR09. (**A**) Slow delayed rectifier K^+^ current (I_Ks_) block through decreased maximal conductance (parameter G_Ks_) at fast (2 Hz) and slow (0.2 Hz) pacing. (**B**) L-type Ca^2+^ current (I_CaL_) enhancement through increased channel permeability (parameter G_CaL_) at fast (2 Hz) and slow (0.2 Hz) pacing.

Reverse rate dependence occurs when a perturbation that changes APD does so to a greater extent at a slow heart rate than at a fast. In other words, as the heart rate slows, the degree of change to the APD increases. Reverse rate dependence has been observed experimentally in response to both several APD altering drugs and to injection of current [12]–[13], and has also been seen in simulation studies [16]. We first confirmed that RRD AP prolongation can be produced in ventricular cell models when parameters representing ion channel properties are altered. Figure 1 shows two examples simulated with the LR09 guinea pig model [17]: decreased slow-delayed rectifier maximal conductance (parameter G_Ks_, Figure 1A), and increased L-type Ca^2+^ channel maximal conductance (parameter G_CaL_, Figure 1B). As expected, both perturbations (a decrease in repolarizing current and an increase in depolarizing current, respectively), prolong the AP. In either case, this prolongation also displays clear reverse rate dependence: the AP prolongation is greater at slow pacing (0.2 Hz) than at fast (2 Hz) for each degree of perturbation, in both absolute and in percent change. Similar effects can be produced in all models that were tested.

### Parameter sensitivities in populations of models identify RRD and FRD perturbations

**Figure 2:**
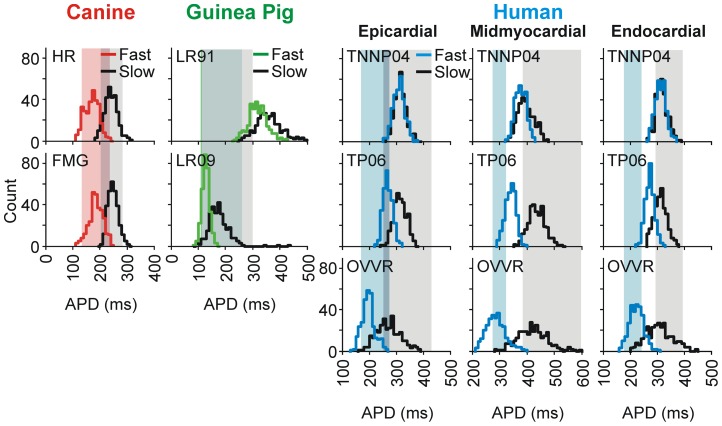
Rate dependence action potential duration across a population of models. APD distributions for models paced at 2(colored histograms) and 0.2 Hz (black histograms). Shaded regions represent experimental ranges estimated from the following sources: canine [11], [55], guinea pig [12], [56]–[57], and human [20], [58]–[61]. Colored shading indicates the range at 2 Hz, gray shading the range at 0.2 Hz, and an intermediate color the range overlap.

In order to understand RRD behavior more comprehensively, we took a systematic approach to analyze the influence of the dozens of parameters that characterize the expression levels and gating of the ion channels in ventricular cell models. We performed a parameter sensitivity analysis, as described previously [15] and in Methods, that allowed us to examine this multitude of parameters in concert and to extract the influence of each on the APD. For each ventricular cell model, we generated a virtual population of hundreds of model variants, in which each individual is defined by a randomly varied set of parameter values. We simulated each model variant at both a fast pacing rate of 2 Hz and a slow pacing rate of 0.2 Hz. We applied this analysis to 7 independent ventricular cell models: TNNP04 [18], TP06 [19], OVVR [20], FMG [21], HR [22], LR91 [23], and LR09 [17] (abbreviations defined in Methods). Additionally, in the human models (TNNP04, TP06, and OVVR), we conducted the analysis on each of the 3 transmural tissue layers: epicardial, mid-myocardial, and endocardial. In all, 13 ventricular myocyte formulations were tested. Distributions of APD across the virtual populations, and a comparison with experimental APD data, are displayed in Figure 2. This shows that faster pacing led to shorter APs in most models, with the notable exceptions of LR91 and TNNP04. This also shows that some models, such as HR, FMG, and OVVR, did notably well in matching the experimentally-observed range of APD.

**Figure 3:**
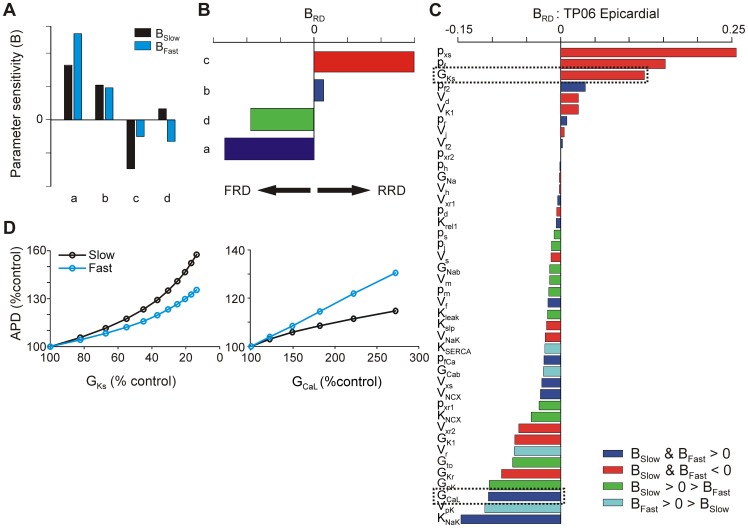
Action potential parameter sensitivity rate dependence. (**A**) Sensitivities (*B*) for hypothetical parameters *a–d* indicate how much each parameter influences the APD. *Black bars* represent that parameter's influence at slow pacing, and *blue bars* its influence at fast pacing. (**B**) B_RD_ is calculated from sensitivities *B* for each parameter in (**A**) as described in *Methods*. Positive B_RD_ indicates a parameter that lengthens the APD with reverse-rate dependence (parameter *c*), negative B_RD_ indicates a parameter that lengthens the APD with forward rate dependence (parameters *a* and *d*), and near-zero B_RD_ indicates neutral rate dependence (parameter *b*). (**C**) B_RD_ for parameters in the TP06 model, derived from parameter sensitivities calculated from a population of 600 virtual myocytes. (**D**) Single perturbation simulation results for parameters G_Ks_ and G_CaL_ (slow delayed rectifier K^+^ channel and L-type Ca^2+^ channel conductance, respectively). Decreasing G_Ks_ increases the APD with reverse rate dependence, and increasing G_CaL_ increases the APD with forward rate dependence, as predicted by each parameter's B_RD_ (C).

To quantify the rate dependence of how the model parameters influence APD, we performed multivariable regression analysis on the population data to statistically relate the model parameters to the simulation outputs. The accuracy of this linear approximation in describing the population behavior has been previously demonstrated [15], [24]–[26] and is quantified in Table 1, which shows R^2^ values resulting from the each model's regression analysis. This analysis generated two sensitivity values for each parameter, B_fast_ and B_slow_, characterizing that parameter's influence on the APD at the two rates. If the effect of a perturbation on APD is rate-dependent, the corresponding parameter sensitivity will differ with rate. We summarize this by calculating B_RD_, which indicates the difference between the parameter sensitivities at the two rates. Figure 3A illustrates some hypothetical possibilities. If the two parameter sensitivities are approximately equal (example b), then B_RD_ is close to zero and changes in the parameter are essentially neutral with respect to rate. On the other hand, positive B_RD_ indicates an RRD perturbation: altering that parameter changes the APD more at a slow rate (example c). Conversely, negative B_RD_ indicates an FRD perturbation: changing that parameter affects the APD more at a fast rate (example a). Finally, example d is a parameter for which B_fast_ and B_slow_ have opposite signs. This indicates that perturbing the parameter will prolong the AP at one rate but shorten it at the other. As discussed in Methods, this behavior does not necessarily fit the RRD/FRD paradigm, but it does represent a perturbation that can lengthen the APD at fast pacing but not at slow. Thus we categorize such parameters with the FRD parameters by assigning a negative B_RD_. For a given ventricular cell model, the B_RD_ plot summarizes the rate dependencies of that model's parameters (Figure 3B).

**Table 1:** Coefficient of determination (R^2^) for linear regression models.

Model	R^2^, Slow	R^2^, Fast
**FMG**	0.9839	0.9035
**HR**	0.9103	0.8159
**LR09**	0.85241	0.97246
**LR91**	0.9732	0.9817
**TNNP04 epi**	0.99257	0.9945
**TNNP04 mid**	0.9918	0.9864
**TNNP04 endo**	0.9934	0.99466
**TP06 epi**	0.99619	0.9929
**TP06 mid**	0.9944	0.97871
**TP06 endo**	0.9969	0.99229
**OVVR epi**	0.9853	0.9882
**OVVR mid**	0.9355	0.9878
**OVVR endo**	0.9773	0.9917

For each model and pacing rate, the accuracy of the linear regression is quantified by the coefficient of determination (R^2^) between the action potential duration values obtained through numerical integration (action potential model) and those computed by matrix multiplication (regression model). The mean R^2^ is 0.968 with a range of 0.816–0.997.

Figure 3C shows the results of this analysis in the TP06 epicardial model. Some parameters are strongly RRD, as expected, but, surprisingly, the majority of parameters are mildly FRD, and some are even strongly FRD. Simulations in which one parameter was varied at a time validate B_RD_ as a quantification of the rate-dependence of a parameter. For example, Figure 3D shows that decreasing G_Ks_ prolongs the AP with reverse rate dependence, as predicted by that parameter's large, positive B_RD_, whereas increasing G_CaL_ prolongs the APD with forward rate dependence, as predicted by that parameter's large, negative B_RD_. B_RD_ was further validated as a measure of rate dependence through single-variable simulations of perturbations to other parameters (Figure S1).

### Capacity for reverse and forward rate-dependence is highly model-dependent

**Figure 4:**
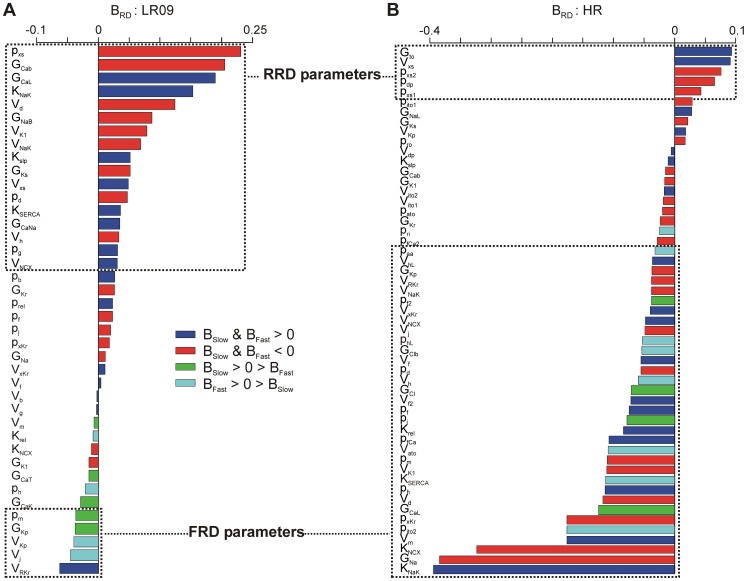
Inter-model comparison of APD parameter sensitivity rate dependence. Ventricular cell models were found to vary greatly in rate dependencies, which is apparent from variations in B_RD_ plot structure. (**A**) B_RD_ for the LR09 guinea pig model, showing largely RRD parameters. (**B**) B_RD_ for the HR canine model, showing a majority of FRD parameters. B_RD_ plots for all models studied can be found in Figures S1–S13 in Text S2.

The distributions of B_RD_ values varied greatly between models. Most models showed a preponderance of RRD parameters, consistent with the hypothesis that reverse rate dependence is intrinsic to any alteration to the APD [13]–[14]. For example, LR09 has mostly RRD parameters (Figure 4A). Of the parameters that are not RRD, most have small B_RD_ magnitude, indicating minimal rate sensitivity. However, several models have a majority of FRD parameters. For instance, we found that most parameters in the HR model are FRD, including, in contrast to LR09, G_CaL_ (Figure 4B). Furthermore, the parameters K_NCX_ (maximal Na^+^-Ca^2+^ exchange current), G_Na_ (maximal fast Na^+^ conductance), and K_NaK_ (maximal Na^+^-K^+^ pump current) all displayed forward rate dependence in the HR model. Results obtained in all models studied are shown in Figures S1Figures S1–S13 in Text S2.

### Capacity for forward rate dependence is related to the rate-dependence of AP morphology

**Figure 5:**
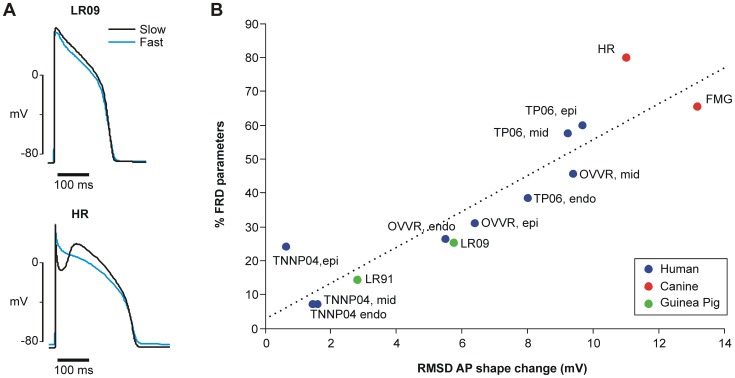
Rate-dependent change in AP contour corresponds to capacity for forward rate-dependence. (**A**) AP traces for a model with mostly RRD parameters (LR09) and a model with mostly FRD parameters (HR). Each trace was calculated after pacing to steady state at each rate. For each model, the fast AP was rescaled with respect to time such that APD_slow_ = APD_fast_, to isolate AP contour changes independent of APD changes. Rate-dependent contour change is quantified by the root mean square deviation (RMSD) between the rescaled AP traces at fast and slow pacing. (**B**) A strong correlation (R^2^ = 0.8366) was observed between the RMSD and the percentage of model parameters that are FRD.

The dramatic differences observed between models led us to wonder what characteristics of the models accounted for these divergent behaviors. We reasoned that if AP shape does not change with rate, then shorter APs at faster rates simply correspond to a faster rate of repolarization. In this case, prior studies have suggested that all perturbations may be inherently RRD [14]. Thus we hypothesized that the number of RRD versus FRD parameters may be related to the changes in AP shape observed with changes in pacing rate. Indeed, we observed that models with larger rate-dependent changes in AP shape have a stronger potential for FRD perturbations, whereas those with minimal rate-dependent changes in AP shape have primarily RRD parameters. For instance, the LR09 model, with almost exclusively RRD parameters (Figure 4A), shows little change in AP shape with rate (Figure 5A) whereas the HR model, with many FRD parameters (Figure 4B), exhibits a substantial change in AP shape with rate (Figure 5A).

To determine if this was generally true across models, we quantified the degree of rate-dependent AP morphology change for each model by calculating the root mean square deviation (RMSD) between AP traces at slow and fast pacing (Figure S2). Before computing RMSD, the simulated AP at the fast rate was rescaled with respect to time to match the duration seen at the slow rate (see Text S1 for details). Each model's capacity for FRD perturbations was quantified by the percent of that model's parameters that are FRD. We found a strong positive correlation (R^2^ = 0.8366) between the RMSD and the percentage of FRD parameters; that is, a greater change in AP shape corresponds to a greater capacity for forward rate dependence (Figure 5B).

We found that the two canine models, HR and FMG, displayed both the highest degree of rate-dependent AP shape change and the highest proportion of FRD parameters. In the HR model, the rate-dependent AP shape change is largely due to the transient outward current (I_to_), which is greater at slow pacing and creates the dramatic phase 1 notch seen at this rate. This effect is present but less prominent in the FMG model. Therefore, we hypothesized that blocking I_to_ in either model would: (1) decrease the rate-dependent AP shape change, and, consequently, (2) reduce the model's capacity for forward rate dependence. We evaluated rate-dependent behaviors in the two models after blocking I_to_ (75% reduction of maximal conductance). This attenuated the phase 1 notch in either model, thereby resulting in a smaller rate-dependent change in AP shape (Figure S3A). This also resulted in a smaller percentage of FRD parameters in either model (Figure S3B). Thus, differences in I_to_ contribute substantially to differences between models.

### Individual parameters exhibit consistency between models

**Figure 6:**
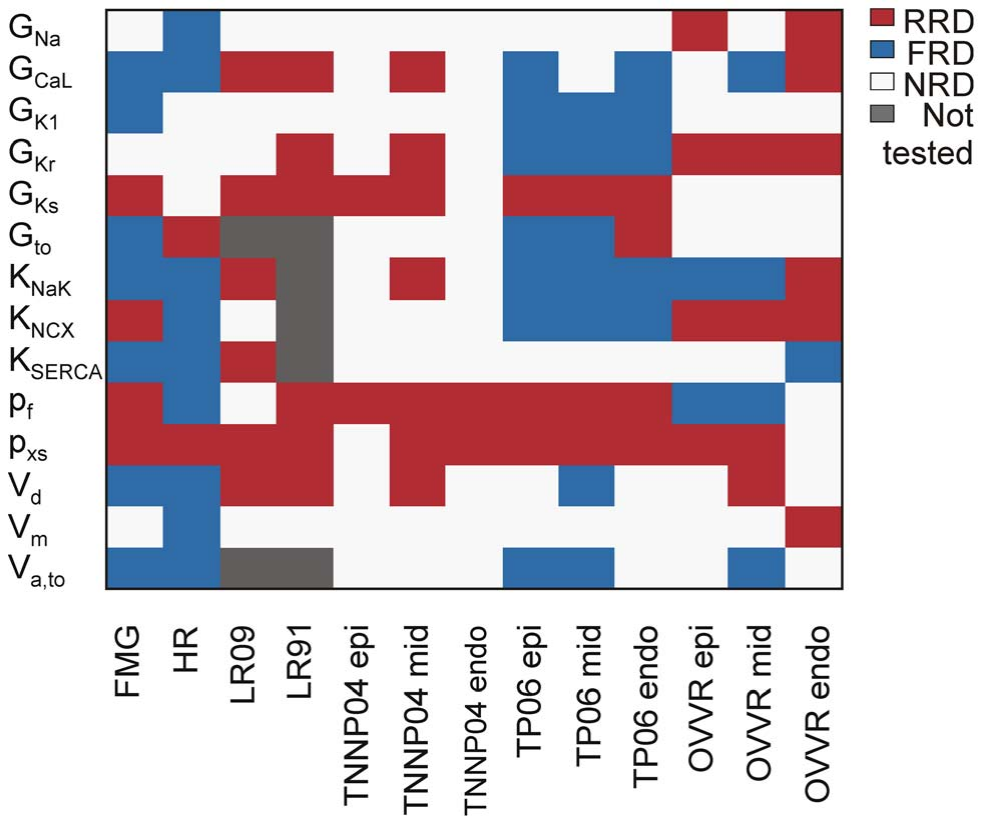
APD parameter sensitivity rate dependence in 13 ventricular myocyte models. For 14 selected parameters that were common to most myocyte models, the heat map shows the rate dependence of these parameters (B_RD_) across the various models. B_RD_≥0.03 is considered reverse rate dependent (*red*), B_RD_≤−0.03 forward rate dependent (*blue*), and −0.03<B_RD_<0.03 non-rate dependent (*white*). *Epi* = epicardial, *mid* = midmyocardial, and *endo* = endocardial.

To examine the consistency of specific predictions across models, we performed two analyses. For these we selected a subset of parameters that are present in almost all models and that correspond to currents generally thought to be important in determining APD. Figure 6 illustrates the rate dependence of these parameters across all the models tested. For this display we do not compare magnitudes of rate dependence across models but instead show whether each parameter is RRD (red), FRD (blue), or non-rate dependent (NRD) (white) in each model. The results show that altering either the magnitude of slow delayed rectifier current (G_Ks_) or the speed of its gating (p_xs_) produces RRD prolongation, suggesting that therapeutic targeting of this current may produce undesirable effects. In contrast, perturbing the magnitudes of either inward rectifier K^+^ current (G_K1_) or the Na^+^-K^+^ pump (K_NaK_) could produce desirable FRD behavior, although this was not true in all models.

For the second analysis we ranked the parameters in each model from the most RRD parameter to the most FRD parameter. We then averaged the relative rank of these parameters across models to generate a rate-dependence consensus list (Table 2). This largely confirms the visual impression of Figure 6, that parameters related to I_Ks_ produce RRD behavior whereas I_K1_ and I_NaK_ may represent legitimate targets for producing FRD prolongation of the AP.

**Table 2:** Parameter rate dependence consensus list.

Parameter	Corresponding current	Average rank
**p_xs_**	I_Ks_	0.13
**G_Ks_**	I_Ks_	0.25
**p_f_**	I_CaL_	0.35
**G_Kr_**	I_Kr_	0.36
**V_d_**	I_CaL_	0.42
**G_CaL_**	I_CaL_	0.52
**K_NCX_**	I_NCX_	0.53
**G_to_**	I_to_	0.55
**G_Na_**	I_Na_	0.56
**K_SERCA_**	SERCA pump	0.56
**V_m_**	I_Na_	0.57
**V_a,to_**	I_to_	0.63
**G_K1_**	I_K1_	0.68
**K_NaK_**	I_NaK_	0.70

To obtain a consensus ranking of rate dependence, parameters within each model were ranked from the most FRD to the most RRD, and this ranking was normalized by the number of parameters per model and averaged across models. Parameter with consensus ranks close to 0 or 1 are consistently RRD or FRD, respectively, across models.

### Simulations uncover mechanisms underlying rate-dependent perturbations

To understand how a given perturbation affects the APD at different rates, we need to examine not only the ionic current directly affected by the perturbation, but all of the other ionic currents in the system. Each current may be indirectly affected by a perturbation, through changes to the AP shape or changes in intracellular ion concentrations, and the sum of their resulting behavior will produce the observed changes in APD.

**Figure 7:**
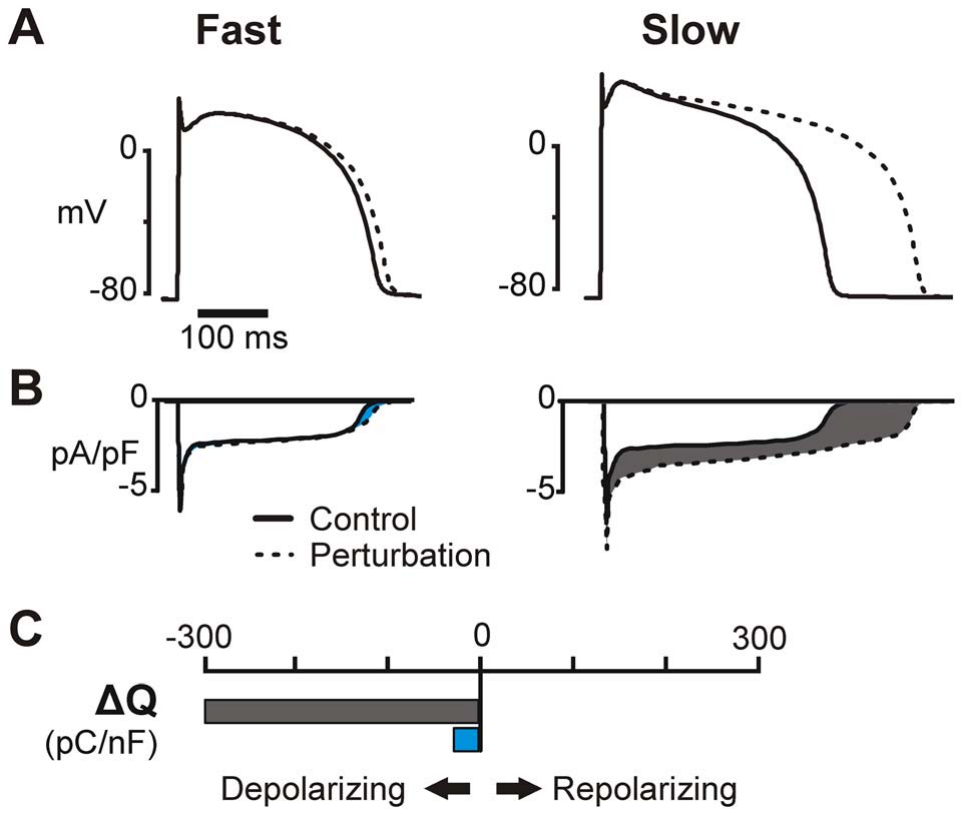
Quantitative contributions of individual ionic currents to AP rate dependence. (**A**) Hypothetical APs before (solid) and after a perturbation that causes RRD prolongation of the AP. (**B**) A specific hypothetical ionic current under the conditions shown in (**A**). This inward (i.e. depolarizing) current increases with the perturbation at the slow rate (gray shaded area) but barely changes at the fast rate (blue shaded area). (**C**) ΔQ is the integral of the difference in current between control and perturbation (shaded areas in (**B**)) in units of pC/nF. The large, negative ΔQ at slow pacing (gray bar) indicates that this current will prolong the AP at this rate, whereas the small, negative ΔQ at fast pacing (blue bar) indicates a minimal alteration of the AP at this rate. This current will therefore contribute to RRD AP prolongation. Figure S4 shows additional hypothetical examples of ΔQ that can contribute to either RRD or FRD behavior.

We developed an analysis method to identify which indirect effects of a perturbation may account for rate dependent AP prolongation. This involved integrating each current from the beginning to the end of the AP under four conditions: with and without the perturbation, at fast and slow rates. The change in the integrated current is denoted ΔQ. If a perturbation changes an integrated current by the same amount at both fast and slow rates, then the current does not contribute substantially to the perturbation's rate dependence. However, if the change in the integrated current varies with rate, then the current may underlie the resulting rate dependence of how the perturbation affects the APD. For example, consider the hypothetical case illustrated in Figure 7. If a perturbation causes a large increase in an inward current, but exclusively at the slow rate, then this current would prolong the APD only at the slow rate, and contribute to reverse rate dependence. Thus by comparing the ΔQ at slow and fast rates, we can determine whether a particular current contributes to rate dependence.

**Figure 8:**
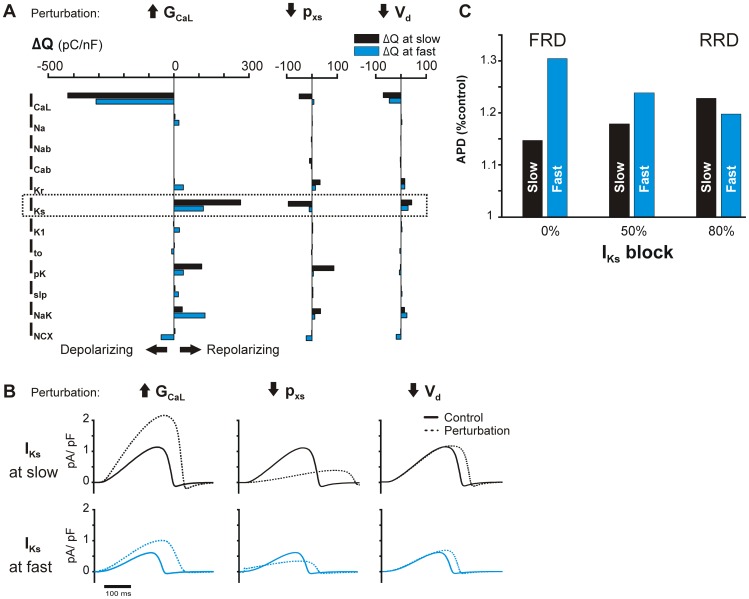
Ionic current changes underlying rate dependence of perturbations in the TP06 epicardial model. (**A**) ΔQ analysis of major ionic currents under 3 perturbations: increase in L-type Ca^2+^ channel maximal conductance, G_CaL_ (271% of baseline, a FRD perturbation), slowed activation of the slow delayed rectifier K^+^ channel (38% of baseline p_xs_, a RRD perturbation), and a negative shift in the voltage dependence of L-type Ca^2+^ activation (5 mV decrease in V_d_, a NRD perturbation). ΔQ quantifies the change in current flux with the perturbation, and is calculated at fast and slow pacing. (**B**) Slow delayed rectifier current (I_Ks_) under each perturbation, at fast and slow pacing. (**C**) APD under increased G_CaL_ (271% baseline) and varying degrees of I_Ks_ block. The APD rate dependence of I_CaL_ enhancement is reversed by simultaneous block of I_Ks,_ as predicted by ΔQ analysis (**A**).

Using this analysis, we were able to identify indirectly-affected currents that are responsible for rate-dependent behavior. In the TP06 epicardial model, we investigated three ionic current perturbations that prolong the APD: an increase in I_CaL_ current density (increased G_CaL_), slowing of I_Ks_ activation (decreased p_xs_), and shifting I_CaL_ activation to more negative membrane potentials (decreased V_d_). All of these changes prolong the APD, but each with a different rate dependence. Increased G_CaL_ produces FRD changes, slower activation of I_Ks_ produces RRD changes, and shifting I_CaL_ activation produces rate-neutral changes. To probe the mechanisms behind these differing rate dependencies, we examined the secondary effects of each perturbation by calculating ΔQ for each current (Figure 8A). This analysis revealed that I_Ks_ in particular displays distinctive rate-dependent behavior with each perturbation, indicating that changes in I_Ks_ contribute to the rate dependence of these specific perturbations. Thus, we examined changes in I_Ks_ time course produced by the different perturbations to gain insight into underlying mechanisms.

Slowing the activation of I_Ks_ (decreasing p_xs_) reduced total I_Ks_ more at slow pacing compared to fast, causing greater AP prolongation at slow than at fast (Figure 8B, middle). This indicates that the RRD effects of slowing I_Ks_ activation depend directly on the rate-dependent behavior of I_Ks_ itself. Shifting the voltage dependence of I_CaL_ activation, a NRD perturbation, caused a small increase in I_Ks_, but the magnitude of the increase was similar at the two rates (Figure 8B, right). Thus, changes in I_Ks_ in this case do not contribute to rate-dependence, consistent with the overall NRD effects of this perturbation. In contrast, increasing G_CaL_ led to an indirect increase in I_Ks_, but with marked rate dependence (Figure 8B, left). Because I_Ks_ increases dramatically more at slow pacing than at fast, this current therefore contributes to the forward rate dependence of enhanced G_CaL_.

We tested the hypothesis that changes in I_Ks_ underlie the forward rate dependence of increased G_CaL_ through a “double perturbation analysis.” This was done by conducting simulations in which we perturbed I_CaL_ as before, but also blocked I_Ks_. Indeed, 50% block of I_Ks_ attenuated the forward rate dependence of I_CaL_ enhancement, and 80% block actually reversed it, resulting in RRD changes to the APD (Figure 8C). Hence, rate-dependent changes in I_Ks_ are critical for the FRD behavior of enhancing G_CaL_.

## Discussion

In this study, we have systematically evaluated rate dependent AP prolongation *in silico*. We analyzed ventricular myocyte models from multiple species and cell types. Our evaluation involved a multivariate approach to parameter sensitivity analysis [15], [24], [26]–[30] that allowed us to both: (1) make global observations in the context of many possible variations, and (2) comprehensively assess all major ionic currents at once. The procedure included a wide range of parameters, representing not only conductances and rates of ion transport but also gating kinetics and voltage dependencies of major ion channels. Through this approach we are able both to generate specific predictions regarding mechanisms of rate-dependent AP prolongation in particular cell types, and to develop more general hypotheses about differences between models and between cell types.

### General principles of AP prolongation uncovered through comparisons between models

At a broad level, a primary finding is that the capacity for forward rate dependence correlates with the extent of rate-dependent changes in the AP contour. Models that exhibit small changes in AP shape with pacing rate have few FRD parameters whereas those that display dramatic changes in AP shape have many more possibilities for FRD perturbations. Further analysis suggests that transient outward current, I_to_, is a major factor underlying the differences between models. For instance, in either the HR or the FMG canine model [21]–[22], blocking I_to_ attenuated, in parallel, both rate dependent changes in AP contour and the number of FRD parameters. Similarly, models of guinea pig myocytes [17], [23], which lack I_to_, tended to show small rate dependent changes in AP shape and to have few FRD parameters. Finally, in all three human models [18]–[20], endocardial cells had both the lowest I_to_ and the fewest FRD parameters compared to mid-myocardial and epicardial myocytes. These observations indicate that the relationship between AP shape change and capacity for forward rate dependence is a general principle, and that I_to_ contributes prominently to differences between models.

The large differences seen between models can both provide new insight into physiological differences and identify discrepancies that need to be resolved, as several recent studies have emphasized [31]–[33]. Most differences between models seem to reflect true physiological differences, such as between canine and guinea pig myocytes, with prominent and minimal I_to_, respectively. On the other hand, differences between models representing the same species and tissue type indicate possible inaccuracies in specific model formulations. These differences can be seen in both global measures of behavior, such as the percentage of FRD parameters, and in specific predictions. For example, block of I_Kr_ had opposite rate-dependent effects in TP06 compared to OVVR. I_Kr_ block was the most RRD perturbation in OVVR (Figures S9–S11 in Text S2), but slightly FRD in TP06 (Figures S6–S8 in Text S2). These findings illustrate that results from a single model should be interpreted cautiously, and they demonstrate the strengths of identifying predictions that are consistent across several models (Figure 6, Table 2, and [26]).

The large set of FRD parameters we identified in several models may appear at odds with the contention that reverse rate dependence is intrinsic [11]–[14]. These results, however, are in fact consistent with and complementary to those predictions. Intrinsic reverse rate dependence has been explained as a consequence of the inverse relationship between net membrane current and APD, whereby a change in membrane current will have a smaller effect on shorter compared with longer APs due to the relative differences in repolarization velocity [11], [14]. In the derivation of this relationship, APD changes with rate, but the AP contour is not otherwise modified. Moreover, simulations to validate intrinsic reverse rate dependence consider the injection of current rather than alterations to any specific ion channels [11], [14]. It is thereby a generic property of the AP. However, this property does not preclude rate dependencies of individual currents or of the effects of perturbations to individual currents. Differing rate-dependent behavior in different currents creates the possibility for non-RRD APD prolongation despite a baseline RRD response. Furthermore, dramatic rate-dependent behavior in individual currents is likely to manifest as rate-dependent changes in the AP contour. It follows that the rate-dependent change in AP contour and the capacity for forward rate-dependence should be strongly correlated, as we found (Figure 5B). We can therefore extend and refine previous ideas [14] by noting that intrinsic reverse rate dependence can in principle be overcome if rate-dependent changes to the AP contour are sufficiently large.

### Specific mechanistic predictions

At a more mechanistic level, our simulations have generated new predictions about how particular perturbations lead to rate-dependent changes in APD. Although these predictions are mostly specific to particular models, they nonetheless offer new mechanistic insight. For instance, we found that enhanced I_CaL_ density produced FRD AP prolongation in the TP06 epicardial model. By systematically examining the behavior of each major current after this perturbation (ΔQ analysis), we were able to identify the indirect, rate-dependent increase in I_Ks_ as the basis of the forward rate dependence (Figure 8). Closer examination of the APs revealed that increased I_CaL_ raises plateau voltage at slow pacing only (Figure S1), resulting in the greatly increased activation of I_Ks_ at slow pacing. While this result may be model-specific, it demonstrates two important points. First, rate-dependent changes in plateau voltage may be an indirect strategy to achieve forward rate dependence via activation of I_Ks_. Second, more generally, a global analysis of parameter sensitivities followed by more fine-grained simulations is an efficient method for elucidating novel and potentially counterintuitive mechanisms [15], [26], [30].

Parameters relating to I_Ks_ were the most consistently RRD across models; slowed activation of current (decreased p_xs_) and smaller maximal conductance (decreased G_Ks_) ranked as the first and second most RRD perturbations, respectively, in a consensus list based on results from all models (Table 2). We investigated slowed I_Ks_ activation in the TP06 model and found that, at slow pacing, this perturbation resulted in considerably smaller peak I_Ks_ and delayed repolarization (Figure 8B). At fast pacing, however, this perturbation caused an immediate rise in I_Ks_ at the beginning of the AP (Figure 8B), and this tempered the effect of the slower activation, resulting in less AP prolongation at this rate. This occurred because slower I_Ks_ activation simultaneously retarded I_Ks_ deactivation. This in turn led, at fast rates, to accumulation of channels in the open state. This particular effect is similar to rate-dependent changes in I_Ks_ that are proposed to occur in some [9], [34] but not all species [35]–[37]. These observations, combined with the known role of I_Ks_ in repolarization reserve [26], [38]–[39] and its heightened importance in repolarization during β-adrenergic stimulation [38], [40]–[43], warrant caution when targeting this current therapeutically.

### Experiments to gain additional insight suggested by these simulations

The substantial capacity for forward rate dependence seen in this study appears to conflict with experimental data examining a wide range of pharmacologic agents in multiple species, which support the conclusion that reverse rate dependence is an almost universal response to diverse APD-modifying perturbations [11]–[13]. As noted above, rate-dependent changes in AP contour are the key determinant of differences in forward rate dependence capacity between models. Consistent with this idea, in the study that proposed intrinsic reverse rate dependence [11], canine AP recordings show little change in shape with pacing rate, and recordings at a slow rate (0.2 Hz) lack the prominent phase 1 notch that we observed at the same pacing rate in the HR and FMG models. A possible reason for the discrepancy could therefore be that experimental recordings were primarily from endocardial myocytes whereas the HR [22] and FMG [21] models were developed to represent epicardial and midmyocardial myocytes, respectively. Prior experimental studies show that canine epicardial cells exhibit larger rate-dependent changes in AP contour than endocardial cells [44], and our simulations predict that reverse rate dependence would be less universal in these myocytes. These findings point to the need for additional experiments to determine whether the degree of intrinsic reverse rate dependence varies between cells isolated from different transmural layers.

The comparison of particular parameters between models (Figure 6) and the consensus list of parameter rate-dependences (Table 2) suggest additional targets that should be tested to determine whether they produce FRD or RRD AP prolongation. In particular, the simulations suggest that inhibition of either I_K1_ or I_NaK_ can produce FRD behavior in several models. The high rank of Na^+^-K^+^ ATPase is consistent with recent studies demonstrating the importance of this current in determining rate-dependent AP shortening in ventricular myocytes [20], [45], as recently reviewed [46], but the potential for therapeutic interventions to produce beneficial AP prolongation has not been thoroughly explored. These examples again illustrate how a systematic analysis of models can suggest novel and perhaps unexpected experimental tests.

### Limitations and future work

The limitations of this study offer suggestions for future research that can provide additional insight into rate-dependent AP prolongation. One limitation is that our regression-based sensitivity analysis implicitly assumes that linear relationships between parameters and model outputs are reasonably accurate. Although our calculations demonstrated that this approximation describes the behavior of our virtual populations quite well (Table 1), non-linear effects may need to be considered when simulating either particular parameter combinations or more extreme changes to parameters. A second limitation is that we examined isolated cell models at steady-state pacing. This strategy allowed us to elucidate a fundamental relationship between the AP contour and the propensity for FRD or RRD changes. To examine how perturbations influence the rate-dependence of proarrhythmia biomarkers such as dispersion of repolarization between transmural layers, simulations would have to be performed with a multicellular model. For instance, Sadrieh et al. used a similar sensitivity analysis approach to examine how ionic conductances influence metrics derived from a simulated pseudo-electrocardiogram [47]; this strategy can be extended to search for perturbations that affect tissue-level biomarkers with desirable rate dependence.

A third limitation is that we did not consider the effects of β-adrenergic stimulation, which almost always accompanies a physiological increase in heart rate. In addition to increasing heart rate via its effects on the sinoatrial node, β-adrenergic stimulation modulates several ionic currents in ventricular cells [43], [48]. This alters the rate dependence of the APD in a manner that is dependent on AP contour and the rate dependencies of specific underlying currents [49]–[50]. Therefore, the rate-dependent effects of at least some perturbations, particularly those that alter I_Ks_ and I_CaL_, are likely to change in the context of β-adrenergic stimulation.

Fourth, we have modeled each perturbation as either a decrease in a maximum rate of ion transport, a voltage-independent change in gating kinetics, or a shift in steady-state activation or inactivation along the voltage axis. We have not considered the fact that the interaction of a drug with a channel may itself be rate dependent due to the fact that drugs bind to some channel states more strongly than to other states [51]–[52]. In future studies it would be interesting to investigate how these effects conspire with the cellular-level effects we have identified to make particular drugs either more FRD or more RRD.

Despite these limitations, our study provides novel insight into the clinically-relevant phenomenon of RRD AP prolongation in ventricular myocytes. FRD prolongation can be produced if a perturbation causes indirect effects to other ionic currents that have appropriate rate-dependent properties. Additionally, a myocyte's capacity for FRD prolongation depends on the extent to which its AP contour changes with rate. More generally, the study illustrates the benefits of the computational approach we have taken. As this work and other recent studies have shown, significant advantages can be gained by evaluating parameter sensitivities thoroughly [47], [53]–[54] and by comparing results from multiple models [33], [52]. Examining models comprehensively provides a wealth of predictions and allows for the identification of counterintuitive behaviors. Moreover, systematic comparisons between models can provide novel insights into mechanisms underlying divergent behaviors. Such modeling strategies will undoubtedly lead to additional breakthroughs in the future.

## Methods

### Models and software

We conducted the rate-dependent parameter sensitivity analysis in 7 ventricular cell models, representing human, canine, and guinea pig myocytes. The complete set of models examined is as follows: ten Tusscher, Noble, Noble & Panfilov (TNNP04) [18], ten Tusscher & Panfilov (TP06) [19], O'Hara, Virag, Varro, & Rudy, (OVVR) [20], Fox, McHarg, & Gilmour (FMG) [21], Hund & Rudy (HR) [22], Luo & Rudy Phase 1 (LR91) [23], and Livshitz & Rudy (LR09) [17]. Additionally, in each human model (TNNP04, TP06, and OVVR), we conducted the analysis on each of the 3 myocardial transmural tissue layers: epicardial, mid-myocardial, and endocardial. Model formulation changes that differentiate tissue layers include several parameter baseline values and channel gating variables (see Tables S6c, S7c, and S8c in Text S1). All models were implemented in MATLAB (The MathWorks, Natick, MA) and numerically integrated using a solver for stiff systems (ode15s).

### Stimulation protocol

At the beginning of each simulation, the cell was allowed to rest for 30–60 s before the stimulus was applied at regular intervals (2 Hz or 0.2 Hz) until steady state was reached. Pacing rate and model formulation could have dramatic effects on the number of stimuli required to reach steady state. Text S1 describes the algorithms that were used to determine steady state conditions in each model. Table S1 in Text S1 describes how many stimuli were necessary to reach steady state conditions in each model.

### Multivariable regression analysis

To determine the influence of each parameter on the action potential duration (APD), we performed a multivariable regression analysis, as described elsewhere [15], [25]. For each model at each pacing rate, we ran 300 trials, each with a different set of randomly varied parameters. Three categories of parameters were varied: (1) maximum conductance and rates of ion transport (G and K), (2) “p” values that scale gating time constants, and (3) voltage shifts, which determine the voltage dependencies of gating. Definitions of all parameters and baseline values can be found in Tables S2–S8 in Text S1. For parameter types “G”, “K”, and “p,” each random set was generated by multiplying the baseline value of each parameter by a log-normally distributed pseudorandom scale factor. The scale factors had median of 1 and the log-transformed scale factors had a standard deviation (σ) of 0.1 (FMG, LR91, TNNP04, TP06) or 0.1823 (HR, OVVR, LR09), meaning that about 95% of parameters are between 82–122% (σ = 0.1) or 70–144% (σ = 0.1823) of control. Parameter type “V”, is an additive rather than a multiplicative factor, so each pseudorandom set was generated from a normal distribution with a mean of 0 and a standard deviation of 2 mV. Trials that displayed AP alternans, APs that failed to repolarize, or an APD more than 3 standard deviations from the mean were removed from the population before regression analysis. From each population of 300, we removed an average of 3.38 trials (range = 0–25).

The regression analysis generates a matrix of parameter sensitivities, **B**, such that changes in model outputs (**Y**) can be approximated as the change in parameters (**X**) times **B** (i.e., **Ŷ** = **XB**≈**Y**). For each parameter, if **B** is positive, increasing the parameter prolongs the APD, and if **B** is negative, decreasing it prolongs the APD. **B_fast_** and **B_slow_** are the parameter sensitivities calculated at fast and slow pacing. When the regression is initially performed, parameter sensitivities are defined relative to the standard deviation of the log-transformed output, i.e. the variability in APD across the simulated population. In order to directly compare results obtained with fast and slow pacing, we scale the values in **B_fast_** by the ratio of the two output standard deviations. In other words, each value is multiplied by σ_APD,fast_/σ_APD,slow_, and this allows for a direct comparison between the parameter sensitivities obtained at the two rates.

### Rate dependence of parameter sensitivity and AP morphology

A vector that we call **B_RD_** quantifies the rate dependence how each parameter influences APD. If **B_slow_** and **B_fast_** have the same sign,
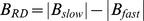
(1)Parameters for which |**B_slow_**|>|**B_fast_**| have a larger effect on the APD at slow pacing, and thus are RRD and are assigned a positive B_RD_. Parameters for which |**B_slow_**|<|**B_fast_**| have a larger effect on the APD at fast pacing, and thus are FRD and are assigned a negative **B_RD_**.

If **B_slow_** and **B_fast_** have opposite signs,
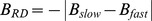
(2)All parameters with opposite signs of B_slow_ and B_fast_ are assigned a negative B_RD_ value because they can be perturbed in such a way as to lengthen the APD at fast but not at slow pacing. For example, if B_fast_ is positive and B_slow_ is negative, increasing this parameter will lengthen the APD at fast pacing and shorten it at slow. Conversely, if B_fast_ is negative and B_slow_ is positive, decreasing this parameter will lengthen the APD at fast pacing and shorten it at slow. In either case, it is possible to achieve the desired outcome of preferential AP prolongation at fast pacing.

The threshold for rate dependence was set at 0.01, so B_RD_>0.01 was considered RRD, B_RD_<−0.01 was considered FRD, and −0.01 ≤B_RD_≤0.01 was defined as NRD.

To quantify rate-dependent AP morphology change, we calculated the root mean square deviation (RMSD) between the AP time-course at fast and at slow pacing for each model, as described in Text S1. AP time courses were obtained under baseline, steady-state conditions. To directly compare the shorter APs obtained at fast rates with the longer APs seen at slow rates, fast AP time courses were rescaled with respect to time prior to the RMSD calculation. The two waveforms compared therefore had identical APD.

#### Calculation of parameter rate dependence consensus rank

For each model, parameters were ranked based on B_RD_. First, each parameter was given an integer rank from 1 to the number of parameters, starting from the highest B_RD_ value (the most RRD) to the lowest (the most FRD). This was then normalized by the number of parameters in that model, so the most FRD parameter had a ranking of 1, and the most RRD parameter had a value close to 0. To calculate consensus values, the average of the normalized ranks was calculated for each parameter (Table 2).

#### Calculation of ΔQ

To understand the mechanisms of rate-dependence in AP prolongation, we need to examine both the direct and indirect effects of perturbation. Each perturbation, or variation in a parameter, will affect the APD both directly, through changes in the current governed by that parameter, and indirectly, through changes in other currents produced by differences in membrane potential or intracellular ion concentrations. To determine which indirect effects of a perturbation may account for rate-dependent prolongation of the AP, we integrated each of the underlying currents from the beginning to the end of the AP under four conditions: with and without the perturbation, at slow and at fast pacing. This integral, termed Q, represents the amount of electrical charge carried by a particular current over the course of the AP.
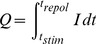
(3)Positive Q indicates net outward current, and negative Q indicates net inward current. For a given perturbation, we calculated ΔQ, the amount by which the integrated current changes with that perturbation:
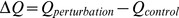
(4)Currents with positive ΔQ contribute to a decrease in APD whereas currents with negative ΔQ contribute to an increase in APD. For instance, positive ΔQ would be calculated from an outward current that increases in magnitude with a perturbation, and this change would contribute to membrane repolarization and a shorter APD. In contrast, negative ΔQ would be calculated from an outward current that gets smaller with a perturbation, thereby contributing to AP prolongation. Additional examples are illustrated in Figures 7 and S4. If a perturbation changes an integrated current by the same amount at fast and slow rates, that current is considered neutral, i.e. not contributing to rate dependence. However, if a perturbation changes an integrated current by different amounts at different rates, that is, ΔQ changes with rate, the current may contribute to the rate dependent effects of the perturbation. We used this analysis to determine ionic currents contributing to different rate-dependence behaviors in the model simulations (Figures 7 and 8).

## Supporting Information

Figure S1 **Validation of specific parameter rate dependence categorization in TP06 epicardial model.** (**A**) B_RD_ of parameters p_xs_, G_Ks_, V_d_, G_Kr_, and G_CaL_. (**B**) APs when each parameter in (A) is individually perturbed at slow (0.2 Hz) and fast (2 Hz) pacing. (**C**) APD as a function of the degree of perturbation for each simulation in (**B**).
(TIF)

Figure S2 **Rate dependence of AP contour in 13 ventricular myocyte models.** (**A**) Steady-state AP traces at slow (0.2 Hz, black) and fast (2 Hz, cyan) pacing under baseline conditions. The fast AP is rescaled with respect to time such that APD_slow_ = APD_fast_ to isolate AP contour changes independent of APD. (**B**) Root mean square deviation (RMSD, in mV) calculated from each pair of APs in (**A**).
(TIF)

Figure S3 **I_to_ block in canine models reduces rate-dependent AP shape change and forward rate dependence capacity.** (**A**) Steady state AP traces at slow (0.2 Hz, black) and fast (2 Hz, cyan) pacing under baseline conditions (left) and after 75% I_to_ block (right). AP traces are rescaled with respect to time as in Figures 4 and S15. Rate-dependent AP shape change is reduced by I_to_ block in both models. (**B**) Parameter sensitivity analysis of canine models under I_to_ block demonstrates a reduction in the capacity for forward rate dependence, consistent with the linear relationship defined by other models (non-I_to_ block data points and linear fit repeated from Figure 5).
(TIF)

Figure S4 **Illustration of calculation of quantitative contributions of individual currents to AP rate dependence.** Possible responses of a hypothetical, specific current to a perturbation at fast and slow pacing and the corresponding ΔQ. (**A**) Current responses to a perturbation that contribute to RRD APD-prolongation include an increase in inward current (*I1*) or decrease in outward current (*I2*) that is greater at slow pacing, or a decrease in inward current (*I3*) or increase in outward current (*I4*) that is greater at fast pacing. (**B**) Current responses to a perturbation that contribute to FRD APD-prolongation include a decrease in inward current (*I5*) or increase in outward current (*I6*) that is greater a slow pacing, or an increase in inward current (*I7*) or decrease in outward current (*I8*) that is greater at fast pacing. (**C**) Current responses that do not change with rate (*I9–I12*) do not contribute to rate-dependent APD-prolongation.
(TIF)

Text S1 **Supplemental methods and tables.**
(DOC)

Text S2 **Parameter sensitivity values (B) and rate dependence (B_RD_) in 13 ventricular myocyte models.** (**Figure S1 in Text S2**) LR91. (**Figure S2 in Text S2**) LR09. (**Figure S3 in Text S2**) TNNP04 epicardial. (**Figure S4 in Text S2**) TNNP04 midmyocardial. (**Figure S5 in Text S2**) TNNP04 endocardial. (**Figure S6 in Text S2**) TP06 epicardial. (**Figure S7 in Text S2**) TP06 midmyocardial. (**Figure S8 in Text S2**) TP06 endocardial. (**Figure S9 in Text S2**) OVVR epicardial. (**Figure S10 in Text S2**) OVVR midmyocardial. (**Figure S11 in Text S2**) OVVR endocardial. (**Figure S12 in Text S2**) HR. (**Figure S13 in Text S2**) FMG.
(DOC)
