# Supplementary material for: Comprehensive Analyses of Ventricular Myocyte Models Identify Targets Exhibiting Favorable Rate Dependence
Source: PLoS Comput Biol. 2014 Mar 27;10(3):e1003543. doi: 10.1371/journal.pcbi.1003543 (PMC3967944; doi:10.1371/journal.pcbi.1003543)
Supplement: Text S1 — Supplemental methods and tables. (DOC) [file pcbi.1003543.s005.doc]

**SUPPLEMENTAL** **TEXT S1**

***Supplemental methods***

***Calculation of APD***

The APD, the output/biomarker of interest, was defined as APD = t_repolarization_ – t_depolarization_, where t_depolarization_ is the time of max upstroke velocity, and t_repolarization_ is the time when voltage first reaches -60 mV after peak voltage.

***Calculation of RMSD***

To quantify rate-dependent AP morphology change, we calculated the root mean square deviation (RMSD) between the AP time-course at fast and at slow pacing for each model as follows. For each model, we obtained the AP time-course during one beat, under baseline conditions, at the same beat number at which PLS data were collected. To remove APD-dependent differences, we normalized the time-course of the shorter AP to the longer one: t_short,norm_ = t_short_ * APD_long_/APD_short_, and shifted it along the time axis so that both APs started at same t, t_start_. Because of the normalization, both APs also have the same APD and time of repolarization, t_end_. We then calculate the RMSD between the two curves from t_start_ to t_end_:

$RMSD=\sqrt{\frac{1}{t_{end}-t_{start}}\times\int_{t_{start}}^{t_{end}} \left(V_{fast}(t)-V_{slow}(t)\right)^{2}dt}$ (S1)

The integral was calculated numerically using the trapezoidal method (MATLAB function *trapz*).

***Determination of steady-state***

To determine the number of beats to steady state, each model was paced under baseline conditions until the APD varied by less than +/- 1% for 1000 consecutive beats. The first beat in this window was considered the minimum number of beats the model must be paced for all simulations, and was determined separately at fast (2 Hz) and slow (0.2 Hz) pacing.

**Table S1: Beats required to reach steady state**

| **Model** | **Tissue layer** | **Beats to steady state** | | **Number of beats used in simulations** | |
| --- | --- | --- | --- | --- | --- |
|  |  | **Slow** | **Fast** | **Slow** | **Fast** |
| **FMG** |  | 50 | 75 | 200 | 200 |
| **HR** |  | 150 | 1975 | 5000 | 5000 |
| **LR09** |  | 75 | 550 | 800 | 800 |
| **LR91** |  | 1 | 25 | 100 | 100 |
| **OVVR** | epi | 75 | 625 | 700 | 700 |
|  | mid | 25 | 675 | 700 | 700 |
|  | endo | 100 | 550 | 600 | 600 |
| **TP06** | epi | 50 | 575 | 1000 | 1000 |
|  | mid | 75 | 450 | 1000 | 1000 |
|  | endo | 50 | 500 | 1000 | 1000 |
| **TNNP04** | epi | 1 | 25 | 100 | 100 |
|  | mid | 1 | 25 | 100 | 100 |
|  | endo | 1 | 25 | 100 | 100 |

***Model Parameters***

**Table S2**a: Parameters relating to current density in LR91

| Parameter | Definition | Baseline value |
| --- | --- | --- |
| G_Na_ | Maximal Na^+^ conductance | 16 mS/cm^2^ |
| G_si_ | Maximal slow-inward (Ca^2+^) conductance | 0.09 mS/cm^2^ |
| G_K1_ | Inward rectifier current scaling factor^1^ | 0.6047 mS/cm^2^ |
| G_K_ | Delayed rectifier current scaling factor^1^ | 0.282 mS/cm^2^ |
| G_Kp_ | Maximal plateau K^+^ conductance | 0.0183 mS/cm^2^ |
| G_b_ | Maximal background conductance | 0.03921 mS/cm^2^ |

Notes

^1^ Scaling factors for I_K1_ and I_K_ in the Luo & Rudy model are not formally maximal conductances, since these factors are multiplied by √K_o_/5.4 and can therefore be greater than this value. For a constant value of extracellular [K^+^], however, changing this factor serves to scale the current.

**Table S2b: Parameters relating to gating kinetics and voltage dependence in LR**91

| Parameter | Definition |
| --- | --- |
| p_m_ | Na^+^ current activation time constant |
| p_h_ | Na^+^ current fast inactivation time constant |
| p_j_ | Na^+^ current slow inactivation time constant |
| p_d_ | Ca^2+^ current (I_si_) activation time constant |
| p_f_ | Ca^2+^ current (I_si_) inactivation time constant |
| p_n_ | Delayed rectifier activation time constant |
| V_m_ | Na^+^ current activation |
| V_h_ | Na^+^ current fast inactivation |
| V_j_ | Na^+^ current slow inactivation |
| V_d_ | Ca^2+^ current (I_si_) activation |
| V_f_ | Ca^2+^ current (I_si_) inactivation |
| V_n_ | Delayed rectifier K^+^ current activation |
| V_ni_ | Delayed rectifier K^+^ current voltage-dependent block/rectification |
| V_x_ | Inward rectifier K^+^ current voltage-dependent block/rectification |
| V_Kp_ | Plateau K^+^ current activation |

Notes [applicable to p and V variables listed for all models]

The “p” variables scale the corresponding time constants at all membrane potentials by 1/p. Thus, p_m_ = 0.5 makes Na^+^ current activation slower by a factor of 2, but does not change the voltage-dependence of activation. By definition, the default value of all p variables 1.

The “V” variables shift the gating variable “infinity curves” along the voltage axis by a fixed amount without affecting kinetics. Thus V_m_ = +5 means that the curve describing steady-state activation of the Na^+^ current (m_∞_) is shifted to the right by 5 mV. By definition, the default value of all V variables is 0.

**Table S3**a: Parameters relating to current density in LR09

| Parameter | Definition | Baseline value |
| --- | --- | --- |
| G_Na_ | Maximal Na^+^ conductance | 16 mS/μF |
| G_Nab_ | Background Na^+^ conductance | 0.004 mS/μF |
| G_CaL_ | L-type Ca^2+^ current permeability^1^ | 5.4e-4 cm/s |
| G_CaNa_ | Na^+^ permeability of L-type Ca^2+^ channel^1^ | 6.75e-7 cm/s |
| G_CaK_ | K^+^ permeability of L-type Ca^2+^ channel^1^ | 1.93e-7 cm/s |
| G_CaT_ | T-type Ca^2+^ conductance | 0.05 mS/μF |
| G_Cab_ | Background Ca^2+^ conductance | 3.016e-3 mS/μF |
| G_K1_ | Inward rectifier K^+^ current scaling factor^2^ | 0.75 mS/μF |
| G_Kr_ | Rapid delayed rectifier K^+^ current scaling factor^2^ | 0.02614 mS/μF |
| G_Ks_ | Slow delayed rectifier K^+^ current scaling factor^3^ | 0.433 |
| G_Kp_ | Maximal plateau K^+^ conductance | 0.00552 mS/μF |
| K_NaK_ | Maximal Na^+^-K^+^ pump current^4^ | 2.25 μA/μF |
| K_NCX_ | Na^+^-Ca^2+^ exchange current scaling factor^5^ | 2.5e-4 |
| K_rel_ | SR Ca^2+^ release scaling factor^6^ | 0.125 |
| K_slp_ | Maximal sarcolemmal Ca^2+^ pump current^4^ | 1.15 μA/μF |
| K_SERCA_ | Maximal rate of SR Ca^2+^ uptake (SERCA)^7^ | 8.75e-3 mM/ms |

Notes

^1^L-type Ca^2+^ current in the Livshitz & Rudy model is computed using Goldman-Hodgkin-Katz current equation rather than an Ohm’s law approximation. Current magnitude is therefore determined by a permeability, in units of distance/time, rather than a specific conductance. The factors controlling Ca^2+^,Na^+^, and K^+^ current through this channel are respectively referred to as P_Ca_, P_Na_, and P_K_ in the Livshitz & Rudy study. Here these parameters are called G_CaL_, G_CaNa_, and G_CaK_ so that terminology is consistent throughout the manuscript.

^2^The scaling factors for I_K1_ and I_Kr_ are not formally maximal conductances, since each is multiplied by √K_o_/5.4 and can therefore be greater than this value. For a constant value of extracellular [K^+^], changing this factor served to scale the current.

^3^The scaling factor for I_Ks_ is not formally a maximal conductance, since it is multiplied by a function of intracellular [Ca^2+^] and can therefore be greater than this value.

^4^Parameters controlling the magnitudes of Na^+^-K^+^ pump current and sarcolemmal Ca^2+^ pump current are all expressed as maximal current densities in units of μA/μF. The variable names used in the Livshitz & Rudy manuscript, are, respectively, Ī_NaK_ and Ī_pCa_. To keep terminology consistent, we refer to these parameters here as K_NaK_ and K_slp_.

^5^ This scaling factor for the Na^+^-Ca^2+^ exchange current is named termed c1 in the Livshitz & Rudy manuscript.

^6^ This amplitude coefficient of SR Ca^2+^ release is termed α_rel_ in the Livshitz & Rudy manuscript.

^7^ In the Livshitz & Rudy. model the parameter Ī_up_, in units of mM/ms, describes the maximal uptake through the SR Ca^2+^ pump. This is now termed K_SERCA_ to be consistent with other parameters.

**Table S3**b: Parameters relating to gating kinetics and voltage dependence in LR09

| Parameter | Definition |
| --- | --- |
| p_m_ | Na^+^ current activation time constant |
| p_h_ | Na^+^ current fast inactivation time constant |
| p_j_ | Na^+^ current slow inactivation time constant |
| p_d_ | L-type Ca^2+^ current activation time constant |
| p_f_ | L-type Ca^2+^ current voltage-dependent inactivation time constant |
| p_b_ | T-type Ca^2+^ current activation time constant |
| p_g_ | T-type Ca^2+^ current inactivation time constant |
| p_xKr_ | Rapid delayed rectifier K^+^ current activation time constant |
| p_xs_ | Slow delayed rectifier K^+^ current activation time constant |
| p_release_ | SR Ca2+ release current from JSR to myoplasm time constant |
| V_m_ | Na^+^ current activation |
| V_h_ | Na^+^ current fast inactivation |
| V_j_ | Na^+^ current slow inactivation |
| V_d_ | L-type Ca^2+^ current activation |
| V_f_ | L-type Ca^2+^ current voltage-dependent inactivation |
| V_b_ | T-type Ca^2+^ current activation |
| V_g_ | T-type Ca^2+^ current inactivation |
| V_xKr_ | Rapid delayed rectifier K^+^ current activation |
| V_xs_ | Slow delayed rectifier K^+^ current activation |
| V_K1_ | Inward rectifier K^+^ current inactivation |
| V_RKr_ | Rapid delayed rectifier K^+^ current rectification |
| V_Kp_ | Plateau K^+^ current activation |
| V_NaK_ | Voltage-dependence of Na^+^-K^+^ pump |
| V_NCX_ | Voltage-dependence of Na^+^-Ca^2+^ exchange current |

**Table S4**a: Parameters relating to current density in FMG

| Parameter | Definition | Baseline value |
| --- | --- | --- |
| G_Na_ | Maximal Na^+^ conductance | 12.8 mS/μF |
| G_Nab_ | Background Na^+^ conductance | 0.0031 mS/μF |
| G_CaL_ | Maximal L-type Ca^2+^ current permeability^1^ | 0.226 μm/ms |
| G_CaK_ | K^+^ permeability of L-type Ca^2+^ current^1^ | 5.79 x10^-3^ μm/ms |
| G_Cab_ | Background Ca^2+^ conductance | 3.84 x10^-4^ mS/μF |
| G_to_ | Maximal transient outward K^+^ conductance | 0.23815 mS/μF |
| G_Kr_ | Rapid delayed rectifier K^+^ current scaling factor^2^ | 0.0136 mS/μF |
| G_Ks_ | Maximal slow delayed rectifier K^+^ conductance | 0.0245 mS/μF |
| G_K1_ | Maximal inward rectifier K^+^ conductance | 2.8 mS/μF |
| G_Kp_ | Maximal plateau K^+^ conductance | 0.002216 mS/μF |
| K_NaK_ | Maximal Na^+^-K^+^ pump current^3^ | 0.693 μA/μF |
| K_NCX_ | Maximal Na^+^-Ca^2+^ exchange current^3^ | 1500 μA/μF |
| K_rel_ | SR Ca^2+^ release scaling factor^4^ | 6 ms^-1^ |
| K_leak_ | Passive SR Ca^2+^ leak scaling factor^4^ | 1 x ^10-6^ ms^-1^ |
| K_SERCA_ | Maximal rate of SR Ca^2+^ uptake (SERCA)^5^ | 0.1 μM/ms |
| K_slp_ | Maximal sarcolemmal Ca^2+^ pump current^3^ | 0.05 μA/μF |

Notes

^1^L-type Ca^2+^ current in the Fox et al. model is computed using Goldman-Hodgkin-Katz current equation rather than an Ohm’s law approximation. Current magnitude is therefore determined by a permeability, in units of distance/time, rather than a specific conductance. The factors controlling Ca^2+^ and K^+^ current through this channel are respectively referred to as $\overline{P}_{Ca}$ and $\overline{P}_{CaK}$in the Fox et al. study. Here these parameters are called G_CaL_ and G_CaK_ so that terminology is consistent throughout the manuscript.

^2^The scaling factor for I_Kr_ is not formally a maximal conductance, since this is multiplied by √K_o_/4 and can therefore be greater than this value. For a constant value of extracellular [K^+^], changing this factor served to scale the current.

^3^Parameters controlling the magnitudes of Na^+^-K^+^ pump current, Na^+^-Ca^2+^ exchange current, and sarcolemmal Ca^2+^ pump current are all expressed as maximal current densities in units of μA/μF. The variable names used in the Fox et al. manuscript, are, respectively, Ī_NaK_, k_NaCa_, and Ī_pCa_. To keep terminology consistent, we refer to these parameters here as K_NaK_, K_NCX_, and K_slp_.

^4^The parameters controlling the maximal rates of SR Ca^2+^ release and passive SR Ca^2+^ leak, termed, respectively, $\overline{P}_{rel}$ and $\overline{P}_{leak}$ in the Fox et al. study, are here called K_rel_ and K_leak_ for consistency.

^5^In the Fox et al. model the parameter V_up_, in units of μM/ms, describes the maximal uptake through the SR Ca^2+^ pump. This is now termed K_SERCA_ to be consistent with other parameters.

Table S4b: Parameters relating to gating kinetics and voltage dependence in FMG

| Parameter | Definition |
| --- | --- |
| p_m_ | Na^+^ current activation time constant |
| p_h_ | Na^+^ current fast inactivation time constant |
| p_j_ | Na^+^ current slow inactivation time constant |
| p_d_ | L-type Ca^2+^ current activation time constant |
| p_f_ | L-type Ca^2+^ current voltage-dependent inactivation time constant |
| p_fCa_ | L-type Ca^2+^ current Ca^2+^-dependent inactivation time constant |
| p_xKr_ | Rapid delayed rectifier activation time constant |
| p_xKs_ | Slow delayed rectifier activation time constant |
| p_xto_ | Transient outward K^+^ current activation time constant |
| p_yto_ | Transient outward K^+^ current inactivation time constant |
| V_m_ | Na^+^ current activation |
| V_h_ | Na^+^ current fast inactivation |
| V_j_ | Na^+^ current slow inactivation |
| V_d_ | L-type Ca^2+^ current activation |
| V_f_ | L-type Ca^2+^ voltage-dependent inactivation |
| V_K1_ | Inward rectifier K^+^ current voltage-dependent block/rectification |
| V_xKr_ | Rapid delayed rectifier K^+^ current activation |
| V_xKs_ | Slow delayed rectifier K^+^ current activation |
| V_xto_ | Transient outward K^+^ current activation |
| V_yto_ | Transient outward K^+^ current inactivation |
| V_RV_ | Rapid delayed rectifier K^+^ current rectification |
| V_Kp_ | Plateau K^+^ current activation |
| V_NaK_ | Voltage-dependence of Na^+^-K^+^ pump |
| V_NCX_ | Voltage-dependence of Na^+^-Ca^2+^ exchange current |

**Table S5**a: Parameters relating to current density in HR

| Parameter | Definition | Baseline value |
| --- | --- | --- |
| G_Na_ | Maximal Na^+^ conductance | 8.25 mS/μF |
| G_NaL_ | Maximal late Na^+^ conductance | 6.5e-3 mS/μF |
| G_CaL_ | Maximal L-type Ca^2+^ current permeability^1^ | 2.43e-4 cm/s |
| G_Cab_ | Background Ca^2+^ current permeability^1^ | 1.99508e-7 cm/s |
| G_K1_ | Maximal inward rectifier K^+^ conductance^2^ | 0.5 mS/μF |
| G_Kr_ | Maximal rapid delayed rectifier K^+^ conductance^2^ | 0.0138542 mS/μF |
| G_Ks_ | Maximal slow delayed rectifier K^+^ conductance ^3^ | 0.02489 mS/μF |
| G_Kp_ | Maximal plateau K^+^ conductance | 2.76e-3 mS/μF |
| G_to_ | Maximal transient outward K^+^ conductance | 0.19 mS/μF |
| G_Clb_ | Background Cl^-^ conductance^4^ | 2.25e-4 mS/μF |
| G_cl_ | Maximal Ca^2+^-dependent transient outward Cl^-^ current permeability^1^ | 4e-7 cm/s |
| K_NaK_ | Maximal Na^+^-K^+^ pump current^5^ | 0.619 μA/μF |
| K_NCX_ | Maximal Na^+^-Ca^2+^ exchange current^5^ | 4.5 μA/μF |
| K_rel_ | SR Ca^2+^ release scaling factor | 3000 ms^-1^ |
| K_slp_ | Maximal sarcolemmal Ca^2+^ pump current ^5^ | 5.75e-2 μA/μF |
| K_SERCA_ | Maximal rate of SR Ca^2+^ uptake (SERCA)^6^ | 4.38e-3 mM/ms |

Notes

^1^L-type Ca^2+^ current, background Ca^2+^ current, and Ca^2+^-dependent transient outward Cl^-^ current in the Hund & Rudy model are computed using Goldman-Hodgkin-Katz current equation rather than an Ohm’s law approximation. Current magnitude is therefore determined by a permeability, in units of distance/time, rather than a specific conductance. The factors controlling these currents are respectively referred to as P_Ca_, P_Ca,b_, and P_Cl_ in the Hund & Rudy study. Here these parameters are called G_CaL_, G_Cab_, and G_Cl_ so that terminology is consistent throughout the manuscript.

^2^The scaling factors for I_K1_ and I_Kr_ are not formally maximal conductances, since each is multiplied by √K_o_/5.4 and can therefore be greater than this value. For a constant value of extracellular [K^+^], changing this factor served to scale the current.

^3^The scaling factor for I_Ks_ is not formally a maximal conductance, since it is multiplied by a function of intracellular Ca^2+^and can therefore be greater than this value.

^4^The units for this maximal conductance are mistakenly listed as uA/uF in the original manuscript.

^5^Parameters controlling the magnitudes of Na^+^-K^+^ pump current, Na^+^-Ca^2+^ exchange current, and sarcolemmal Ca^2+^ pump current are all expressed as maximal current densities in units of μA/μF. The variable names used in the Hund & Rudy manuscript, are, respectively,Ḡ_NaK_ , ν_max_, and Ḡ_pCa_. Ḡ_NaK_ and Ḡ_pCa_ are mistakenly listed as maximal conductances, in units of mS/μF. To keep terminology consistent, we refer to these parameters here as K_NaK_ , K_NCX_, and K_slp_.

^6^In the Hund & Rudy model the parameter Ī_up_, in units of mM/ms, describes the maximal uptake through the SR Ca^2+^ pump. This is now termed K_SERCA_ to be consistent with other parameters.

**Table S5**b: Parameters relating to gating kinetics and voltage dependence in HR

| Parameter | Definition |
| --- | --- |
| p_m_ | Na^+^ current activation time constant |
| p_h_ | Na^+^ current fast inactivation time constant |
| p_j_ | Na^+^ current slow inactivation time constant |
| p_hL_ | Late Na^+^ current inactivation time constant |
| p_d_ | L-type Ca^2+^ current activation time constant |
| p_dp_ | Power to which “d” is raised |
| p_f_ | L-type Ca^2+^ current fast voltage-dependent inactivation time constant |
| p_f2_ | L-type Ca^2+^ current slow voltage-dependent inactivation time constant |
| p_fCa_ | L-type Ca^2+^ current fast Ca^2+^-dependent inactivation time constant |
| p_fCa2_ | L-type Ca^2+^ current slow Ca^2+^-dependent inactivation time constant |
| p_xKr_ | Rapid delayed rectifier activation time constant |
| p_xs1_ | Slow delayed rectifier fast activation time constant |
| p_xs2_ | Slow delayed rectifier slow activation time constant |
| p_ato_ | Transient outward K^+^ current activation time constant |
| p_ito1_ | Transient outward K^+^ current fast inactivation time constant |
| p_ito2_ | Transient outward K^+^ current slow inactivation time constant |
| p_aa_ | Second transient outward K^+^ current activation time constant |
| p_ro_ | SR release current activation time constant |
| p_ri_ | SR release current inactivation time constant |
| V_m_ | Na^+^ current activation |
| V_h_ | Na^+^ current fast inactivation |
| V_j_ | Na^+^ current slow inactivation |
| V_hL_ | Late Na^+^ current inactivation |
| V_d_ | L-type Ca^2+^ current activation |
| V_dp_ | Voltage dependence of the power to which “d” is raised |
| V_f_ | L-type Ca^2+^ current fast voltage-dependent inactivation |
| V_f2_ | L-type Ca^2+^ current slow voltage-dependent inactivation |
| V_K1_ | Inward rectifier K^+^ current voltage-dependent block/rectification |
| V_RKr_ | Rapid delayed rectifier K^+^ current rectification |
| V_xKr_ | Rapid delayed rectifier K^+^ current activation |
| V_xs_ | Slow delayed rectifier K^+^ current activation |
| V_ato_ | Transient outward K^+^ current activation |
| V_ito1_ | Transient outward K^+^ current fast inactivation |
| V_ito2_ | Transient outward K^+^ current slow inactivation |
| V_Kp_ | Plateau K^+^ current activation |
| V_NaK_ | Voltage-dependence of Na^+^-K^+^ pump current |
| V_NCX_ | Voltage-dependence of Na^+^-Ca^2+^ exchange current |

**Table S6**a: Parameters relating to current density in TNNP04

| Parameter | Definition | Baseline value^1^ |
| --- | --- | --- |
| G_Na_ | Maximal Na^+^ conductance | 14.838 mS/μF |
| G_Nab_ | Background Na^+^ conductance | 2.9e-4 mS/μF |
| G_CaL_ | Maximal L-type Ca^2+^ current permeability^2^ | 1.75e-4 cm^3^/μF s^-1^ |
| G_Cab_ | Background Ca^2+^ conductance | 5.92e-4 mS/μF |
| G_to_ | Maximal transient outward K^+^ conductance | 0.294 mS/μF |
| G_Kr_ | Rapid delayed rectifier K^+^ current scaling factor^3^ | 0.096 mS/μF |
| G_Ks_ | Maximal slow delayed rectifier K^+^ conductance | 0.245 mS/μF |
| G_K1_ | Maximal inward rectifier K^+^ conductance^3^ | 5.405 mS/μF |
| G_pK_ | Maximal plateau K^+^ conductance | 0.0146 mS/μF |
| K_NaK_ | Maximal Na^+^-K^+^ pump current^4^ | 1.362 pA/pF |
| K_NCX_ | Maximal Na^+^-Ca^2+^ exchange current^4^ | 1000 pA/pF |
| K_rel1_ | SR Ca^2+^ release scaling factor^5^ | 16.464 μM ms^-1^ |
| K_rel2_ | SR Ca^2+^ release scaling factor^5^ | 8.232 μM ms^-1^ |
| K_leak_ | Passive SR leak scaling factor^5^ | 8e-5 ms^-1^ |
| K_SERCA_ | Maximal rate of SR Ca^2+^ uptake (SERCA) ^6^ | 0.425 μM ms^-1^ |
| K_slp_ | Maximal sarcolemmal Ca^2+^ pump current^4^ | 0.025 pA/pF |

Notes

^1^ For epicardial cells. See “transmural heterogeneity” (table S6c) for differences in endocardial and midmyocardial formulations.

^2^ L-type Ca^2+^ current in the TNNP04 model is computed using the Goldman-Hodgkin-Katz current equation rather than an Ohm’s Law approximation. Current magnitude is therefore determined by a permeability rather than a specific conductance. However this parameter is called G_CaL_ (as in the original study) so that terminology is consistent throughout the manuscript.

^3^ The scaling factors for I_Kr_ and I_K1_ are not formally a maximal conductance, since each is multiplied by √K_o_/5.4 and can therefore be greater than this value. For a constant value of extracellular [K^+^], changing these factors serves to scale the current.

^4^ Parameters controlling the magnitudes of Na^+^-K^+^ pump current, Na^+^-Ca^2+^ exchange current, and sarcolemmal Ca^2+^ pump current are all expressed as maximal current densities in units of pA/pF. The variable names used in the TNNP manuscript are, respectively, P_NaK_, k_NaCa_ and (mistakenly) G_pCa_. To keep terminology consistent, we refer to these parameters here as K_NaK_, K_NCX_, and K_slp_.

^5^ The parameters controlling the maximal rates of SR Ca^2+^ release and passive SR Ca^2+^ leak, termed, respectively, a_rel_, c_rel_, and V_leak_ in the TNNP04 study, are here, for consistency, called K_rel1_, K_rel2_, and K_leak_.

^6^ In the TNNP04 model the parameter V_maxup_, in units of μM/ms, describes the maximal uptake through the SR Ca^2+^ pump. This is now termed K_SERCA_ to be consistent with other parameters.

**Table S6**b: Parameters relating to gating kinetics and voltage dependence in TNNP04

| Parameter | Definition |
| --- | --- |
| p_m_ | Na^+^ current activation time constant |
| p_h_ | Na^+^ current fast inactivation time constant |
| p_j_ | Na^+^ current slow inactivation time constant |
| p_d_ | L-type Ca^2+^ current activation time constant |
| p_f_ | L-type Ca^2+^ current voltage-dependent inactivation time constant |
| p_fCa_ | L-type Ca^2+^ current Ca^2+^-dependent inactivation time constant |
| p_r_ | Transient outward K^+^ current activation time constant |
| p_s_ | Transient outward K^+^ current inactivation time constant |
| p_xs_ | Slow delayed rectifier K^+^ current activation time constant |
| p_xr1_ | Rapid delayed rectifier K^+^ current activation time constant |
| p_xr2_ | Rapid delayed rectifier K^+^ current inactivation time constant |
| p_g_ | Leak current time constant |
| V_m_ | Na^+^ current activation |
| V_h_ | Na^+^ current fast inactivation |
| V_j_ | Na^+^ current slow inactivation |
| V_d_ | L-type Ca^2+^ current activation |
| V_f_ | L-type Ca^2+^ current voltage-dependent inactivation |
| V_r_ | Transient outward K^+^ current activation |
| V_s_ | Transient outward K^+^ current inactivation |
| V_xs_ | Slow delayed rectifier K^+^ current activation |
| V_xr1_ | Rapid delayed rectifier K^+^ current activation |
| V_xr2_ | Rapid delayed rectifier K^+^ current inactivation |
| V_K1_ | Inward rectifier K^+^ current voltage-dependent block/rectification |
| V_NCX_ | Voltage dependence of Na^+^-Ca^2+^ exchange current |
| V_NaK_ | Voltage dependence of Na^+^-K^+^ pump current |
| V_pK_ | Plateau K^+^ current activation |

Transmural heterogeneity

The default parameters represent epicardial (epi) myocytes. To simulate endocardial (endo) and midmyocardial (M) cells, the following parameters are scaled as follows, as per ten Tusscher *et al*.

**Table S6**c: Parameters varied according to cell type

| Parameter | Endo/epi | M/epi |
| --- | --- | --- |
| G_Ks_ | 1 | 0.2531 |
| G_to_ | 0.2483 | 1 |

Additionally, the transient outward K^+^ current inactivation gate (gate s) formulation varies by cell type as follows:

Epi and M:

$$s_{\infty}=\frac{1}{1+e^{(V+20)/5}}$$

$$\tau_{s}=85e^{-(V+45)^{2}/320}+\frac{5}{1+e^{(V-20)/5}}+3$$

Endo:

$$s_{\infty}=\frac{1}{1+e^{(V+28)/5}}$$

$$\tau_{s}=1000e^{-(V+67)^{2}/1000}+8$$

**Table S7**a: Parameters relating to current density in TP06

| Parameter | Definition | Baseline value^1^ |
| --- | --- | --- |
| G_Na_ | Maximal Na^+^ conductance | 14.838 mS/μF |
| G_Nab_ | Background Na^+^ conductance | 2.9e-4 mS/μF |
| G_CaL_ | Maximal L-type Ca^2+^ current permeability^2^ | 3.98e-5 cm/μF ms^-1^ |
| G_Cab_ | Background Ca^2+^ conductance | 5.92e-4 mS/μF |
| G_to_ | Maximal transient outward K^+^ conductance | 0.294 mS/μF |
| G_Kr_ | Rapid delayed rectifier K^+^ current scaling factor^3^ | 0.153 mS/μF |
| G_Ks_ | Maximal slow delayed rectifier K^+^ conductance | 0.392 mS/μF |
| G_K1_ | Maximal inward rectifier K^+^ conductance^3^ | 5.405 mS/μF |
| G_pK_ | Maximal plateau K^+^ conductance | 1.46e-2 mS/μF |
| K_NaK_ | Maximal Na^+^-K^+^ pump current^4^ | 2.724 pA/pF |
| K_NCX_ | Maximal Na^+^-Ca^2+^ exchange current^4^ | 1000 pA/pF |
| K_rel_ | SR Ca^2+^ release scaling factor^5^ | 0.102 ms^-1^ |
| K_leak_ | Passive SR leak scaling factor^5^ | 3.6e-4 ms^-1^ |
| K_SERCA_ | Maximal rate of SR Ca^2+^ uptake (SERCA) ^6^ | 6.375e-3 mM ms^-1^ |
| K_slp_ | Maximal sarcolemmal Ca^2+^ pump current^4^ | 0.1238 pA/pF |

Notes

^1^ For epicardial cells. See “transmural heterogeneity” (table S7c) for differences in endocardial and midmyocardial formulations.

^2^ L-type Ca^2+^ current in the TP06 model is computed using the Goldman-Hodgkin-Katz current equation rather than an Ohm’s Law approximation. Current magnitude is therefore determined by a permeability rather than a specific conductance. However this parameter is called G_CaL_ (as in the original study) so that terminology is consistent throughout the manuscript.

^3^ The scaling factors for I_Kr_ and I_K1_ are not formally a maximal conductance, since each is multiplied by √K_o_/5.4 and can therefore be greater than this value. For a constant value of extracellular [K^+^], changing these factors serves to scale the current.

^4^ Parameters controlling the magnitudes of Na^+^-K^+^ pump current, Na^+^-Ca^2+^ exchange current, and sarcolemmal Ca^2+^ pump current are all expressed as maximal current densities in units of pA/pF. The variable names used in the ten Tusscher & Panfilov manuscript are, respectively, P_NaK_, k_NaCa_ and (mistakenly) G_pCa_. To keep terminology consistent, we refer to these parameters here as K_NaK_, K_NCX_, and K_slp_.

^5^ The parameters controlling the maximal rates of SR Ca^2+^ release and passive SR Ca^2+^ leak, termed, respectively, V_rel_, and V_leak_ in the ten Tusscher & Panfilov study, are here, for consistency, called K_rel_, and K_leak_. Additionally, K_rel_ = 0.102 ms^-1^, as per the source code available at http://www-binf.bio.uu.nl/khwjtuss/ SourceCodes/HVM2/, rather than 40.8 mM ms^-1^ as in the manuscript.

^6^ In the TP06 model the parameter V_maxup_, in units of mM/ms, describes the maximal uptake through the SR Ca^2+^ pump. This is now termed K_SERCA_ to be consistent with other parameters.

**Table S7**b: Parameters relating to gating kinetics and voltage dependence in TP06

| Parameter | Definition |
| --- | --- |
| p_m_ | Na^+^ current activation time constant |
| p_h_ | Na^+^ current fast inactivation time constant |
| p_j_ | Na^+^ current slow inactivation time constant |
| p_d_ | L-type Ca^2+^ current activation time constant |
| p_f_ | L-type Ca^2+^ current voltage-dependent slow inactivation time constant |
| p_f2_ | L-type Ca^2+^ current voltage-dependent fast inactivation time constant |
| p_fCa_ | L-type Ca^2+^ current Ca^2+^-dependent inactivation time constant |
| p_r_ | Transient outward K^+^ current activation time constant |
| p_s_ | Transient outward K^+^ current inactivation time constant |
| p_xs_ | Slow delayed rectifier K^+^ current activation time constant |
| p_xr1_ | Rapid delayed rectifier K^+^ current activation time constant |
| p_xr2_ | Rapid delayed rectifier K^+^ current inactivation time constant |
| V_m_ | Na^+^ current activation |
| V_h_ | Na^+^ current fast inactivation |
| V_j_ | Na^+^ current slow inactivation |
| V_d_ | L-type Ca^2+^ current activation |
| V_f_ | L-type Ca^2+^ current voltage-dependent slow inactivation |
| V_f2_ | L-type Ca^2+^ current voltage-dependent fast inactivation |
| V_r_ | Transient outward K^+^ current activation |
| V_s_ | Transient outward K^+^ current inactivation |
| V_xs_ | Slow delayed rectifier K^+^ current activation |
| V_xr1_ | Rapid delayed rectifier K^+^ current activation |
| V_xr2_ | Rapid delayed rectifier K^+^ current inactivation |
| V_K1_ | Inward rectifier K^+^ current voltage-dependent block/rectification |
| V_NCX_ | Voltage dependence of Na^+^-Ca^2+^ exchange current |
| V_NaK_ | Voltage dependence of Na^+^-K^+^ pump current |
| V_pK_ | Plateau K^+^ current activation |

Transmural heterogeneity

The default parameters represent epicardial (epi) myocytes. To simulate endocardial (endo) and midmyocardial (M) cells, the following parameters are scaled as follows, as per ten Tusscher *et al*.

**Table S7**c: Parameters varied according to cell type

| Parameter | Endo/epi | M/epi |
| --- | --- | --- |
| G_Ks_ | 1 | 0.25 |
| G_to_ | 0.2483 | 1 |

Additionally, the transient outward K^+^ current inactivation gate (gate s) formulation varies by cell type as follows:

Epi and M:

$$s_{\infty}=\frac{1}{1+e^{(V+20)/5}}$$

$$\tau_{s}=85e^{-(V+45)^{2}/320}+\frac{5}{1+e^{(V-20)/5}}+3$$

Endo:

$$s_{\infty}=\frac{1}{1+e^{(V+28)/5}}$$

$$\tau_{s}=1000e^{-(V+67)^{2}/1000}+8$$

**Table S8**a: Parameters relating to current density in OVVR

| Parameter | Definition | Baseline value^1^ |
| --- | --- | --- |
| G_Na_ | Maximal Na^+^ conductance | 75 mS/μF |
| G_NaL_ | Maximal late Na^+^ conductance | 0.0075 mS/μF |
| G_to_ | Maximal transient outward K^+^ conductance | 0.02 mS/μF |
| G_Kr_ | Maximal rapid delayed rectifier K^+^ conductance | 0.046 mS/μF |
| G_Ks_ | Maximal slow delayed rectifier K^+^ conductance | 0.0034 mS/μF |
| G_K1_ | Maximal inward rectifier K^+^ conductance | 0.1908 mS/μF |
| K_NCX_ | Maximal Na^+^-Ca^2+^ exchange current^2^ | 0.0008 μA/μF |
| G_Kb_ | Maximal conductance of background K^+^ | 0.003 mS/μF |
| G_CaL_ | L-type Ca^2+^ current permeability^3^ | 0.0001 cm/s |
| K_NaK_ | Scales the Na^+^-K^+^ ATPase current | 30 |
| G_Nab_ | Background Na^+^ current permeability^3^ | 3.75e-10 cm/s |
| G_Cab_ | Background Ca^2+^ current permeability^3^ | 2.5e-8 cm/s |
| K_SERCA_ | Scales total Ca^2+^ uptake via SERCA pump from myoplasm to NSR | 1 |
| K_rel_ | Scales total Ca^2+^ release via ryanodine receptors from JSR to myoplasm | 1 |

Notes

^1^ For endocardial cells. See “transmural heterogeneity” (table S8c) for differences in epicardial and midmyocardial formulations.

^2^ The parameter controlling the magnitude of the Na^+^-Ca^2+^ exchange current (both myoplasmic and subspace components) is expressed as maximal current density in units of μA/μF. The variable name used in the O’Hara *et al.* manuscript is G_NaCa_. To keep terminology consistent, we refer to this parameter as K_NCX_.

^3^ L-type Ca^2+^ current and background Na^+^ and Ca^2+^ currents in the O’Hara *et al.* model are computed using the Goldman-Hodgkin-Katz current equation rather than an Ohm’s law approximation. Current magnitude is therefore determined by a permeability, in units of distance/time, rather than a specific conductance. The permeabilities of Ca^2+^ current through the L-type channel, background Na^+^, and background Ca^2+^ are respectively referred to as P_Ca_, P_Nab_, and P_Cab_ in the O’Hara *et al.* study. Here these parameters are called G_CaL_, G_Nab_, and G_Cab_ so that terminology is consistent throughout the manuscript.

**Table S8**b: Parameters relating to gating kinetics and voltage dependence in OVVR

| Parameter | Definition |
| --- | --- |
| p_m_ | Fast Na^+^ current activation time constant |
| p_hFast_ | Fast Na^+^ current fast inactivation time constant |
| p_hSlow_ | Fast Na^+^ current slow inactivation time constant |
| p_j_ | Fast Na^+^ current recovery from inactivation time constant |
| p_hL_ | Late Na^+^ current inactivation time constant |
| p_a_ | Transient outward K^+^ current activation time constant |
| p_iFast_ | Transient outward K^+^ current fast inactivation time constant |
| p_iSlow_ | Transient outward K^+^ current slow inactivation time constant |
| p_fFast_ | L-type Ca^2+^ current fast voltage-dependent inactivation time constant |
| p_fSlow_ | L-type Ca^2+^ current slow voltage-dependent inactivation time constant |
| p_fCaFast_ | L-type Ca^2+^ current fast Ca^2+^ -dependent inactivation time constant |
| p_fCaSlow_ | L-type Ca^2+^ current slow Ca^2+^ -dependent inactivation time constant |
| p_jCa_ | L-type Ca^2+^ current recovery from inactivation time constant |
| p_xrFast_ | Rapid delayed rectifier K^+^ current fast activation/deactivation time constant |
| p_xrSlow_ | Rapid delayed rectifier K^+^ current slow activation/deactivation time constant |
| p_xS1_ | Slow delayed rectifier K^+^ current activation time constant |
| p_xS2_ | Slow delayed rectifier K^+^ current deactivation time constant |
| p_xK1_ | Inward rectifier K^+^ current inactivation time constant |
| V_m_ | Na^+^ current activation |
| V_h_ | Na^+^ current inactivation |
| V_mL_ | Late Na^+^ current activation |
| V_hL_ | Late Na^+^ current inactivation |
| V_a_ | Transient outward K^+^ current activation |
| V_d_ | L-type Ca^2+^ current activation |
| V_f_ | L-type Ca^2+^ current voltage-dependent inactivation |
| V_xr_ | Rapid delayed rectifier K^+^ current activation/deactivation |
| V_xs1_ | Slow delayed rectifier K^+^ current activation |
| V_K1_ | Inward rectifier K^+^ current inactivation |

Transmural heterogeneity

The default parameters represent endocardial (endo) myocytes. To simulate epicardial (epi) and midmyocardial (M) cells, the following parameters are scaled as follows, as per O’Hara *et al*.

**Table S8**c: Parameters varied according to cell type

| Parameter | Epi/endo | M/endo |
| --- | --- | --- |
| GNaL | 0.6 | 1 |
| Gto | 4.0 | 4.0 |
| G_CaL_, G_CaNa_, G_CaK_ ^4^ | 1.2 | 2.5 |
| G_Kr_ | 1.3 | 0.8 |
| GKs | 1.4 | 1 |
| GK1 | 1.2 | 1.3 |
| K_NCX_^5^ | 1.1 | 1.4 |
| KNaK | 0.9 | 0.7 |
| GKb | 0.6 | 1 |
| J_rel,NP,∞_, J_rel, CaMK∞_ | 1 | 1.7 |
| J_up,NP_, J_up, CaMK_ | 1.3 | 1 |
| $\left[\overline{CMDN}\right]$ | 1.3 | 1 |

Additionally, the transient outward K^+^ current inactivation gate (gate i) formulation varies by cell type. In epicardial cells, the time constant for both the fast and slow components of the gate (τ_i,fast_ and τ_i,slow_, respectively) are multiplied by the quantity δ_epi_ :

$$\delta_{epi}=1.0-\frac{0.95}{1.0+e^{(V+70.0)/5.0}}$$

Notes

^4^ See note ^3^ above. As G_CaL_ represents L-type channel permeability to Ca^2+^, G_CaNa_ and G_CaK_ represents L-type permeability to Na^+^ and K^+^, respectively.

^5^ See note ^2^ above
