# Supplementary material for: Comprehensive Analyses of Ventricular Myocyte Models Identify Targets Exhibiting Favorable Rate Dependence
Source: PLoS Comput Biol. 2014 Mar 27;10(3):e1003543. doi: 10.1371/journal.pcbi.1003543 (PMC3967944; doi:10.1371/journal.pcbi.1003543)
Supplement: Text S2 — Parameter sensitivity values (B) and rate dependence (BRD) in 13 ventricular myocyte models. (Figure S1 in Text S2) LR91. (Figure S2 in Text S2) LR09. (Figure S3 in Text S2) TNNP04 epicardial. (Figure S4 in Text S2) TNNP04 midmyocardial. (Figure S5 in Text S2) TNNP04 endocardial. (Figure S6 in Text S2) TP06 epicardial. (Figure S7 in Text S2) TP06 midmyocardial. (Figure S8 in Text S2) TP06 endocardial. (Figure S9 in Text S2) OVVR epicardial. (Figure S10 in Text S2) OVVR midmyocardial. (Figure S11 in Text S2) OVVR endocardial. (Figure S12 in Text S2) HR. (Figure S13 in Text S2) FMG. (DOC) [file pcbi.1003543.s006.doc]

**SUPPLEMENTAL** **TEXT S2**


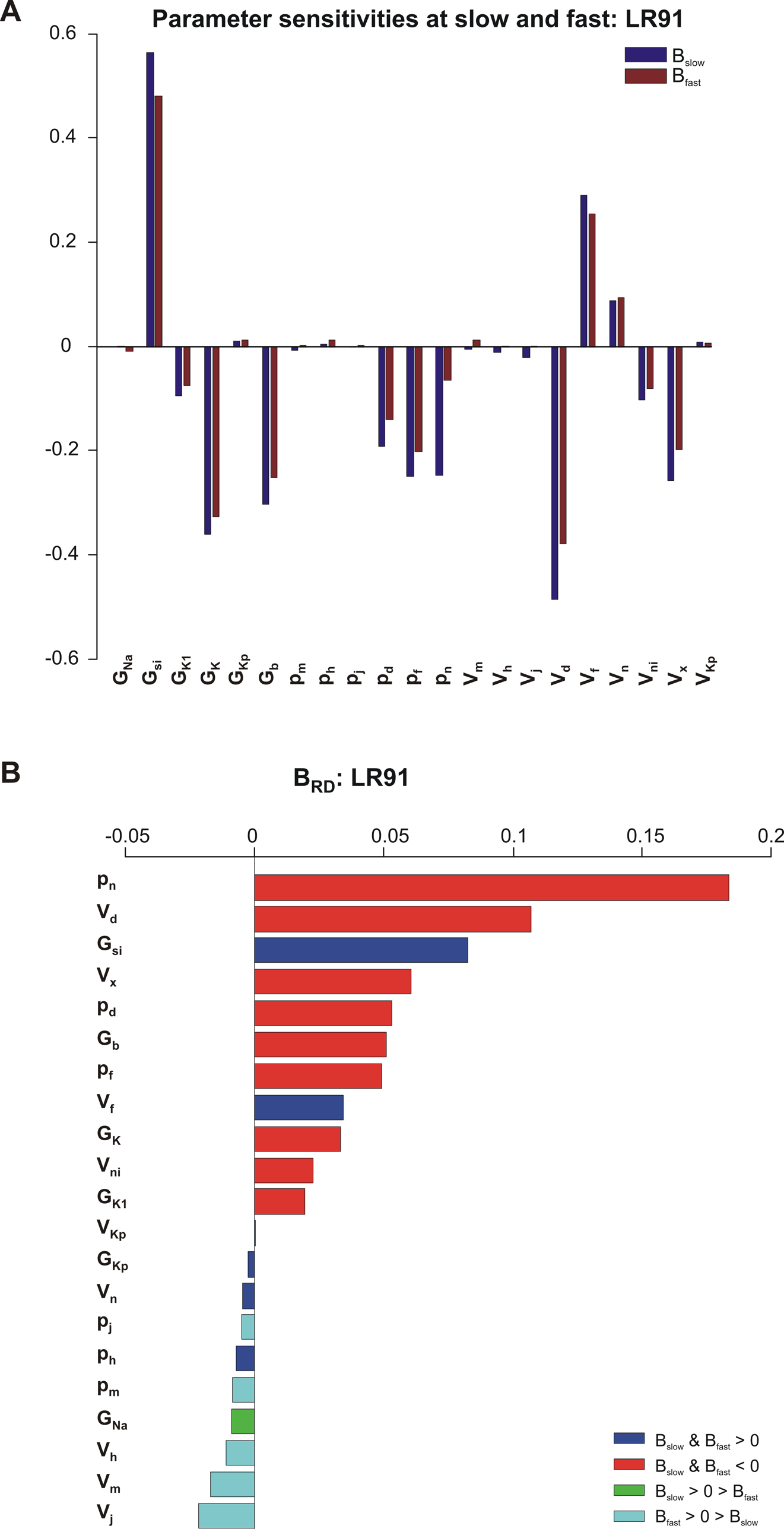


**Figure S1. (A)** Parameter sensitivity values and **(B)** rate dependence in the LR91 model.


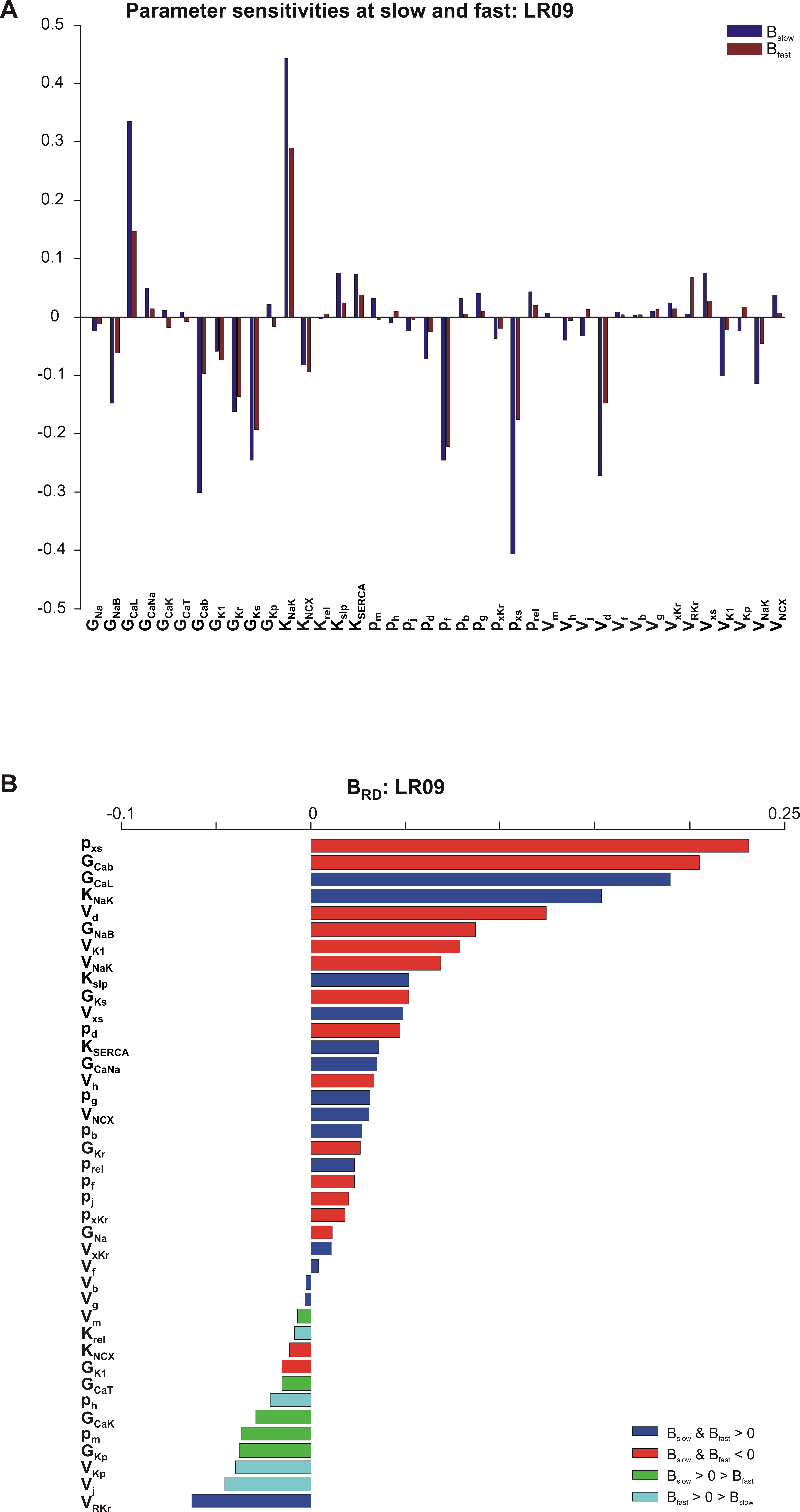


**Figure S2.** **(A)** Parameter sensitivity values and **(B)** rate dependence in the LR09 model.


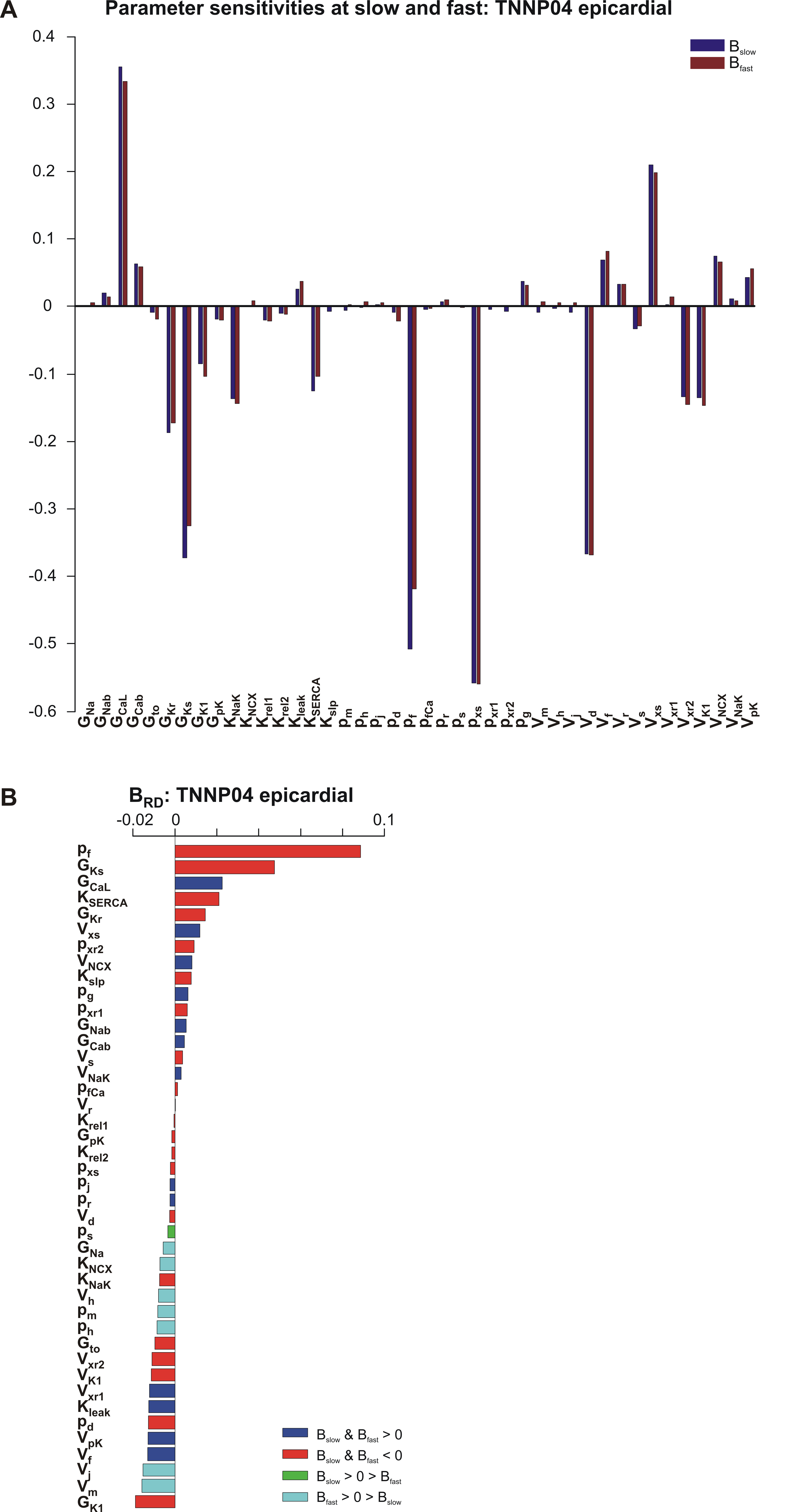


**Figure S3.** **(A)** Parameter sensitivity values and **(B)** rate dependence in the TNNP04 epicardial model.


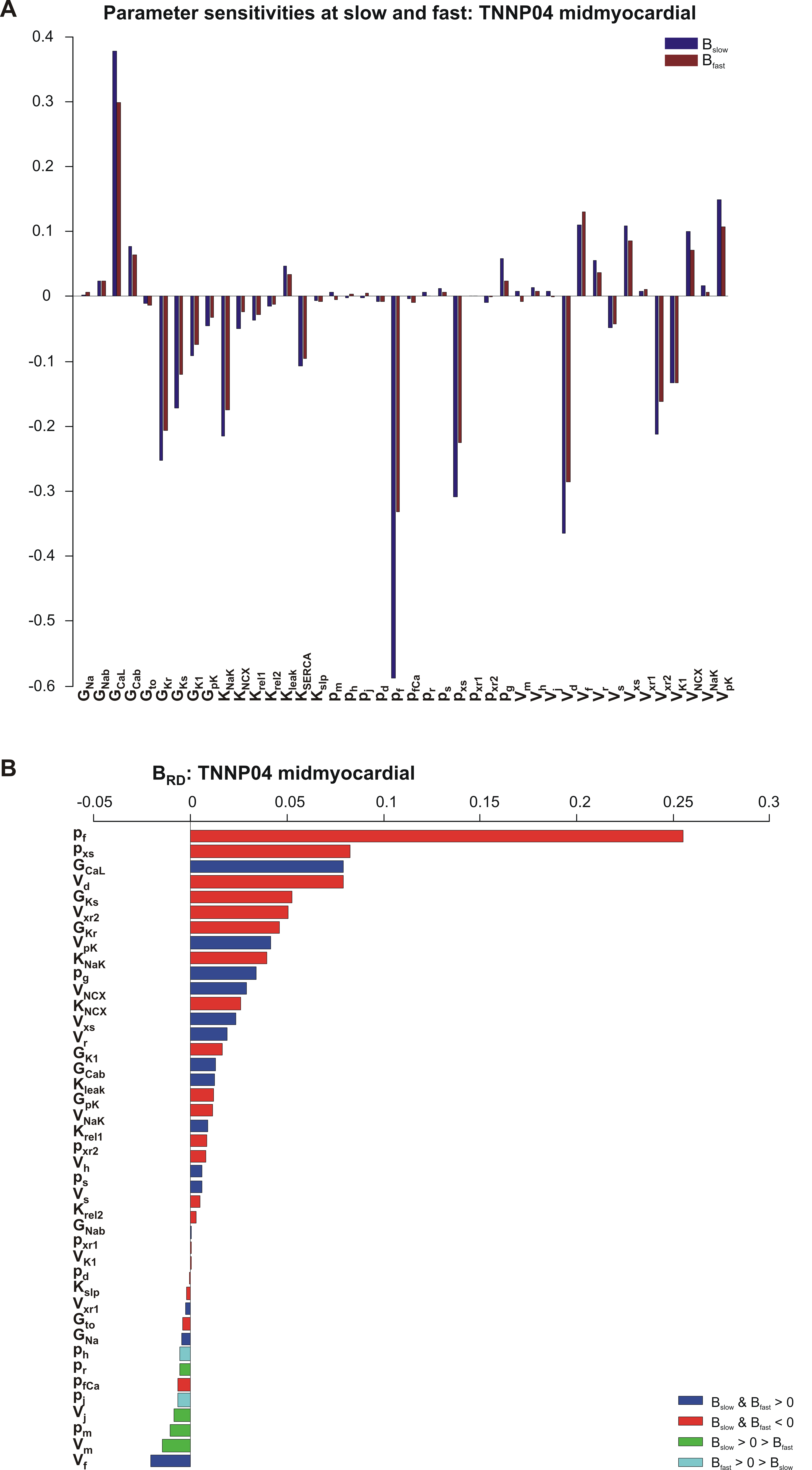


**Figure S4**. (A) Parameter sensitivity values and **(B)** rate dependence in the TNNP04 midmyocardial model.


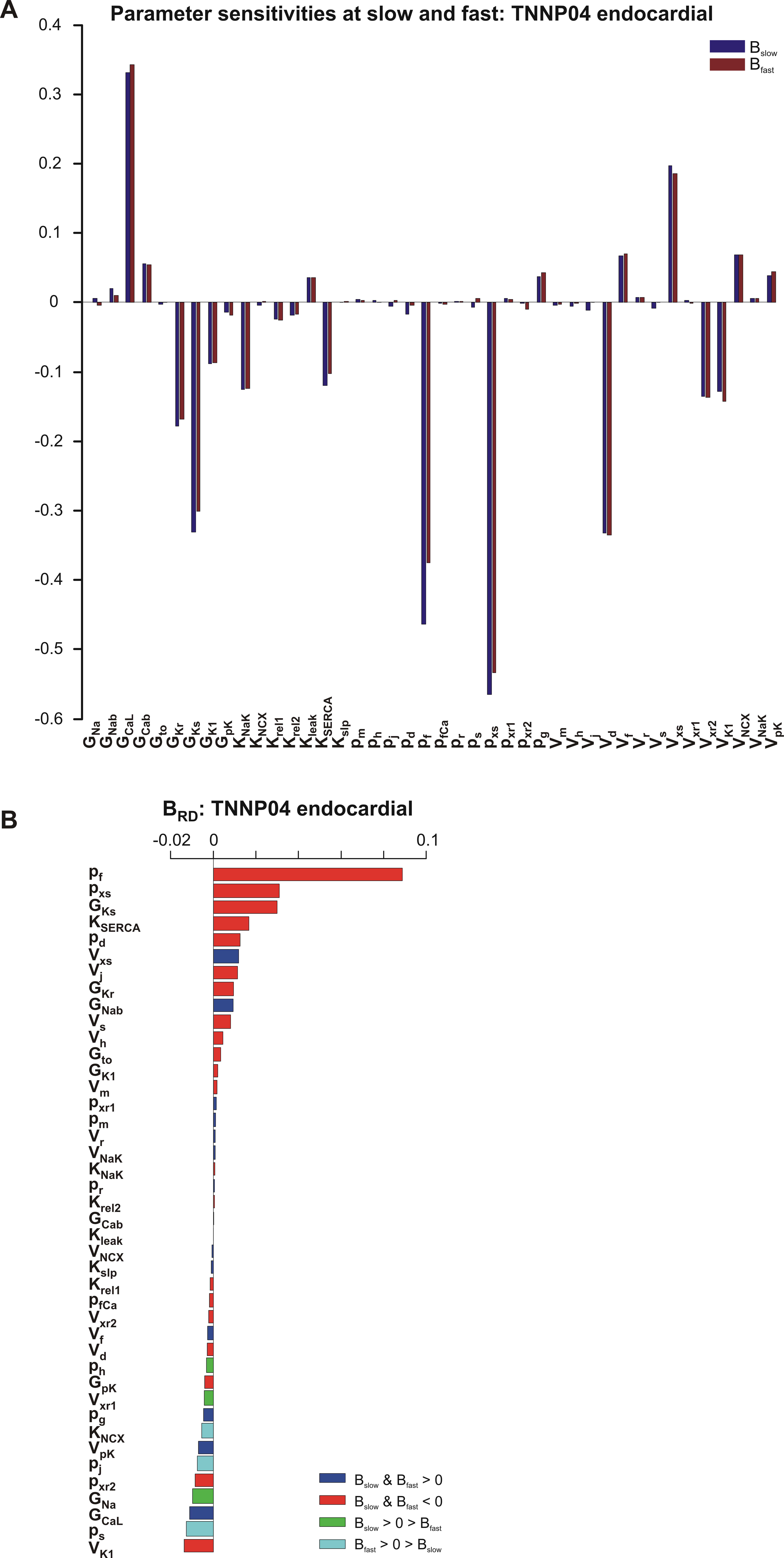


**Figure S5. (A)** Parameter sensitivity values and **(B)** rate dependence in the TNNP04 endocardial model.


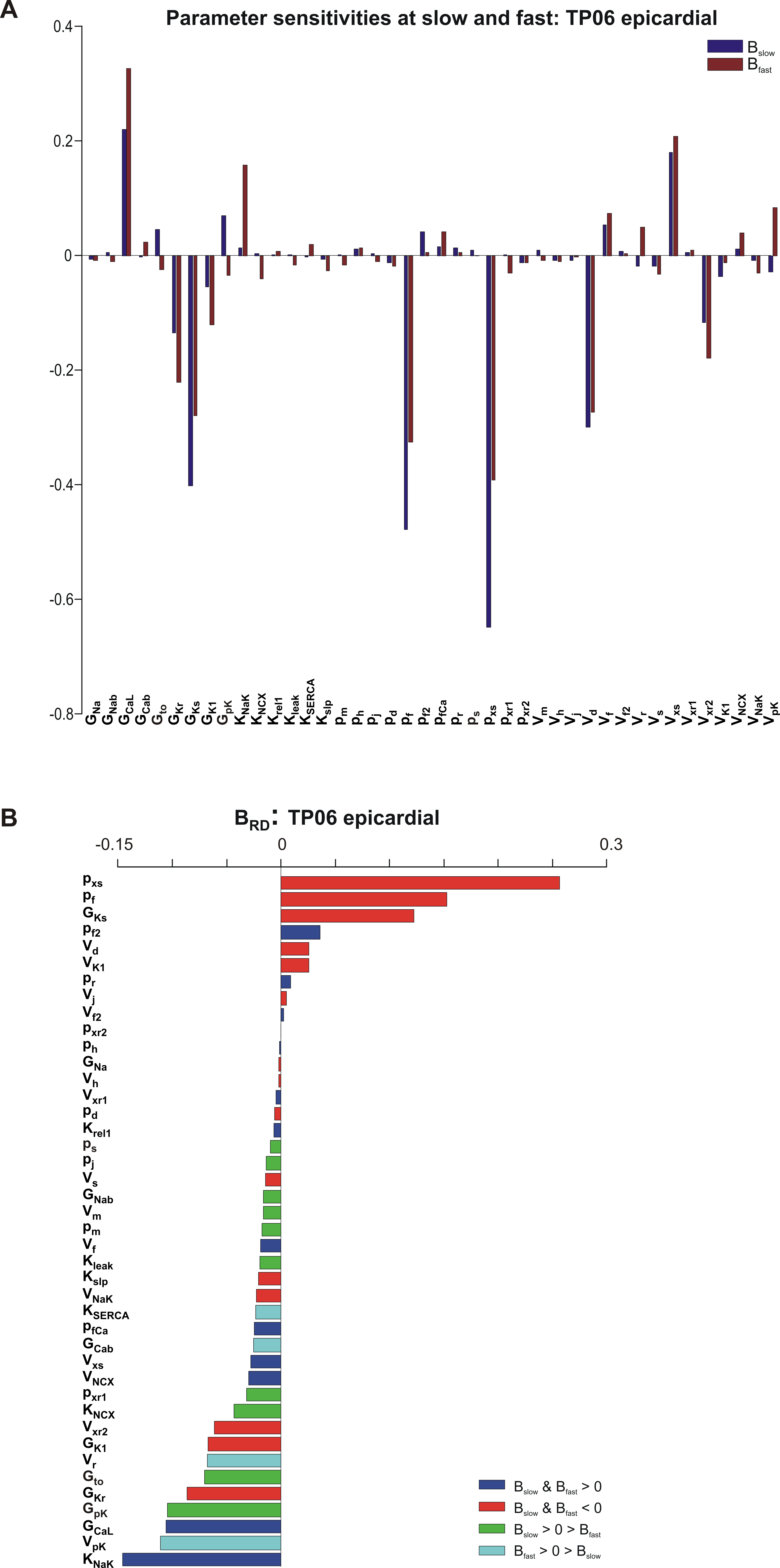


**Figure S6.** **(A)** Parameter sensitivity values and **(B)** rate dependence in the TP06 epicardial model.


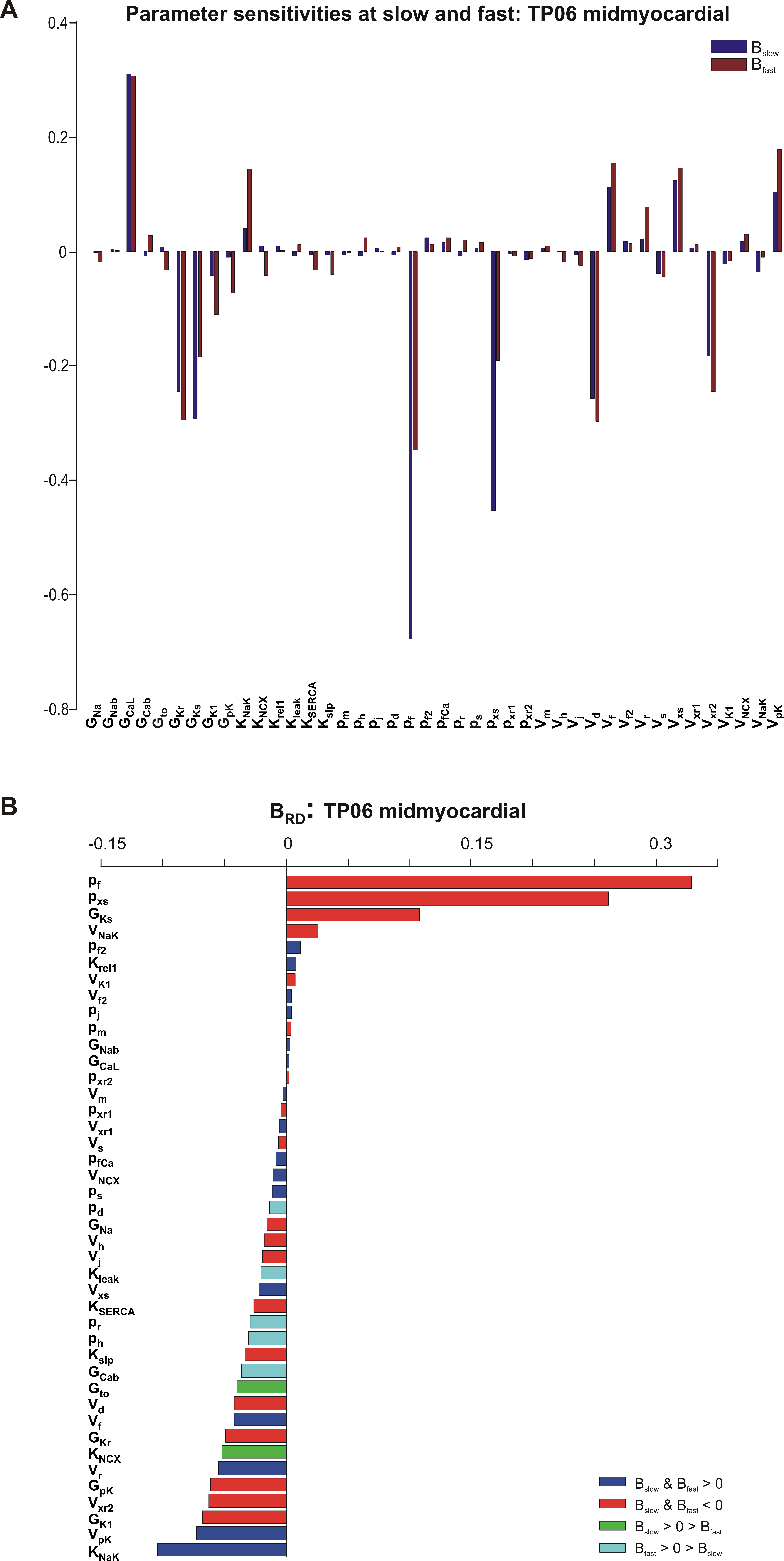


**Figure S7.** **(A)** Parameter sensitivity values and **(B)** rate dependence in the TP06 midmyocardial model.


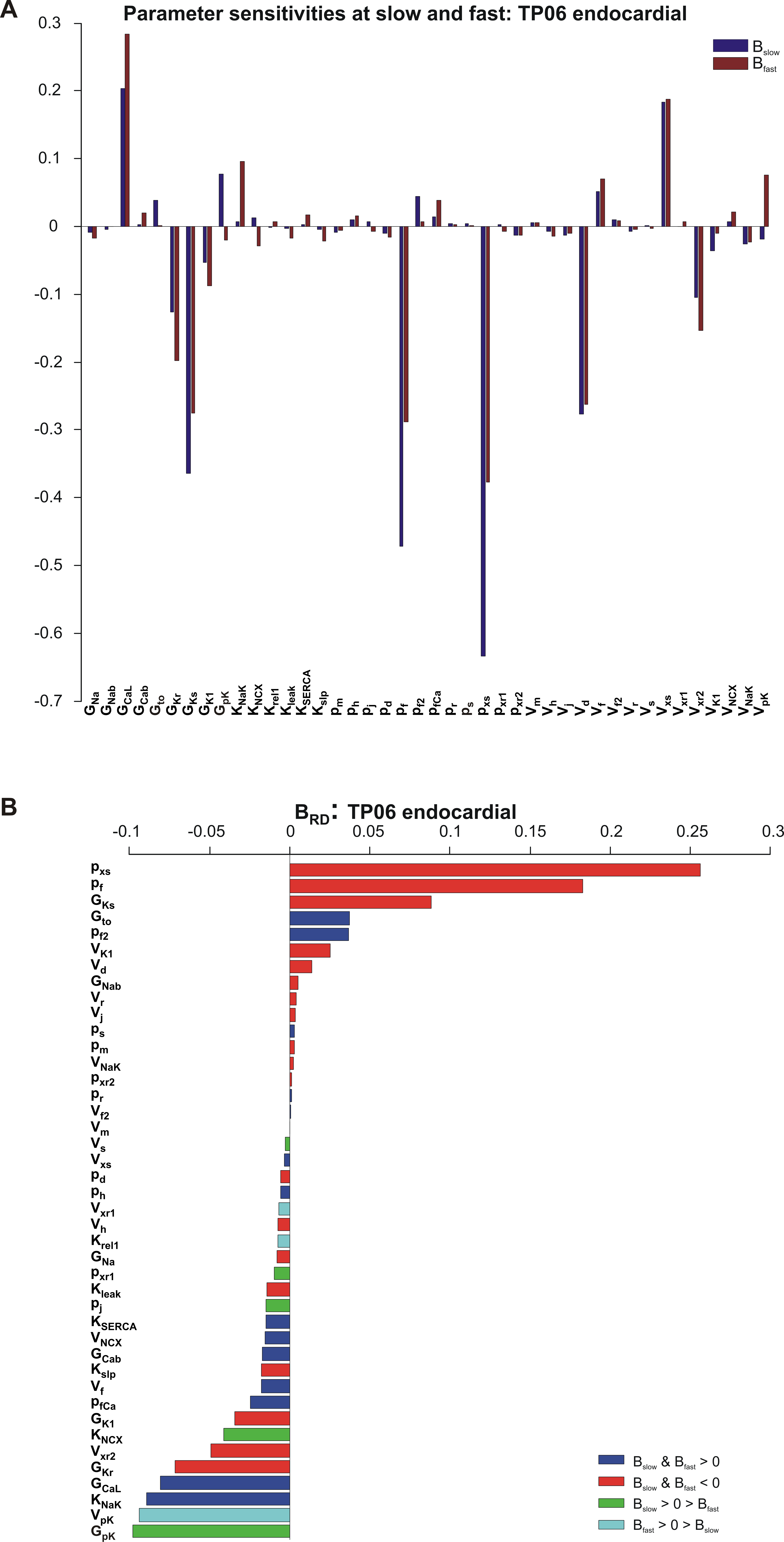


**Figure S8.** **(A)** Parameter sensitivity values and **(B)** rate dependence in the TP06 endocardial model.


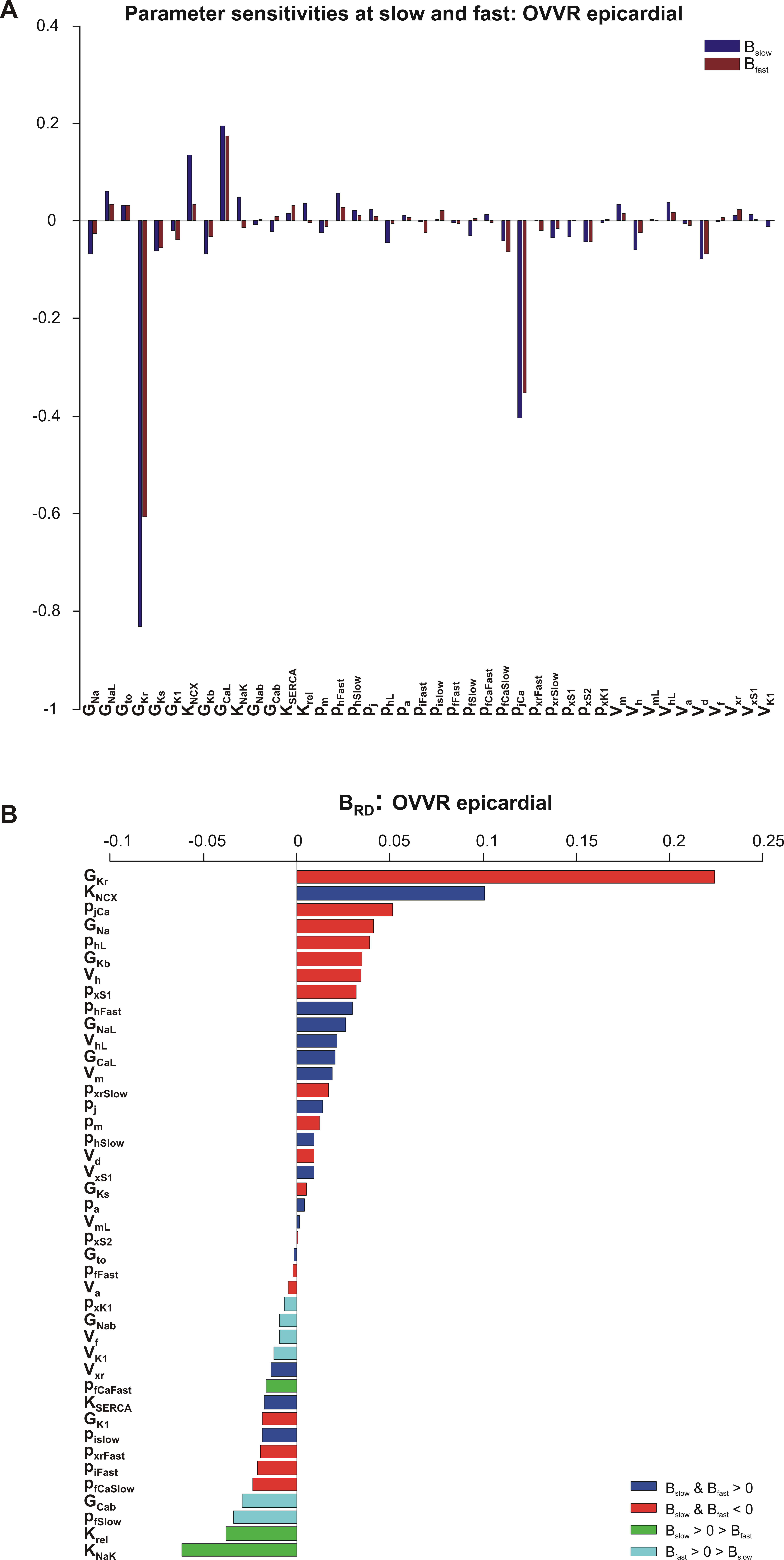


**Figure S9.** **(A)** Parameter sensitivity values and **(B)** rate dependence in the OVVR epicardial model.


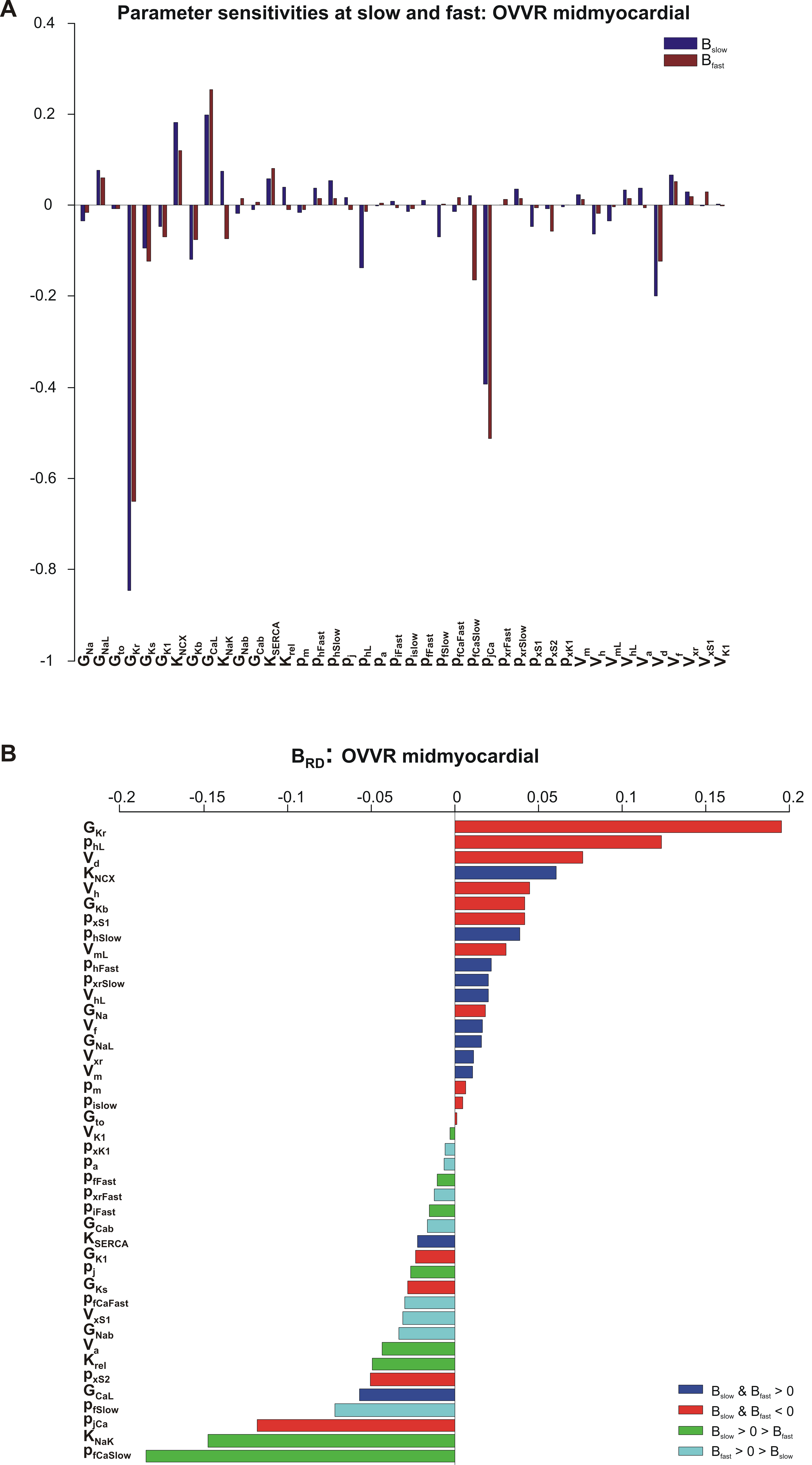


**Figure S10.** **(A)** Parameter sensitivity values and **(B)** rate dependence in the OVVR midmyocardial model.


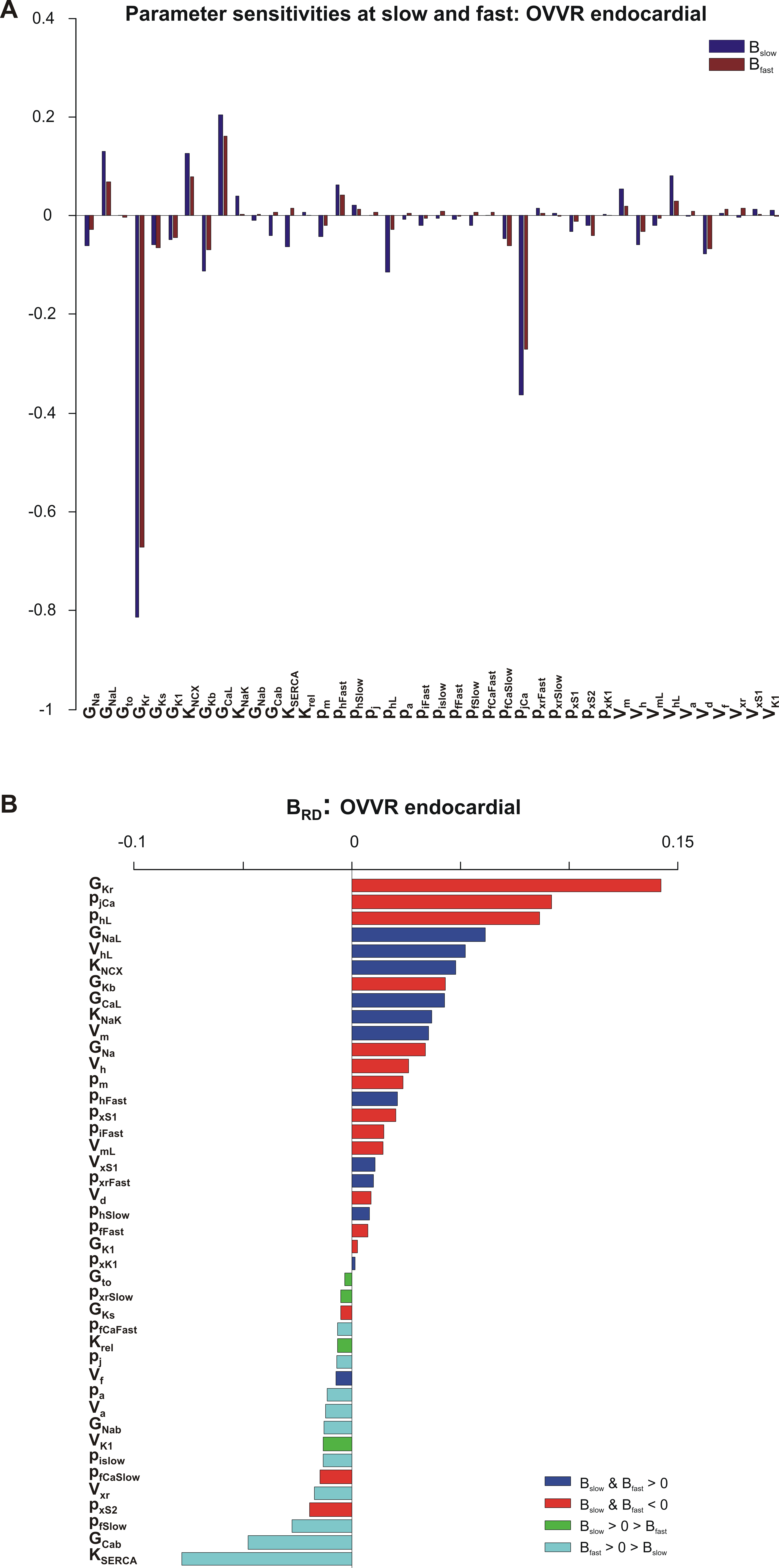


**Figure S11.** **(A)** Parameter sensitivity values and **(B)** rate dependence in the OVVR endocardial model.





**Figure S12.** **(A)** Parameter sensitivity values and **(B)** rate dependence in the HR model.


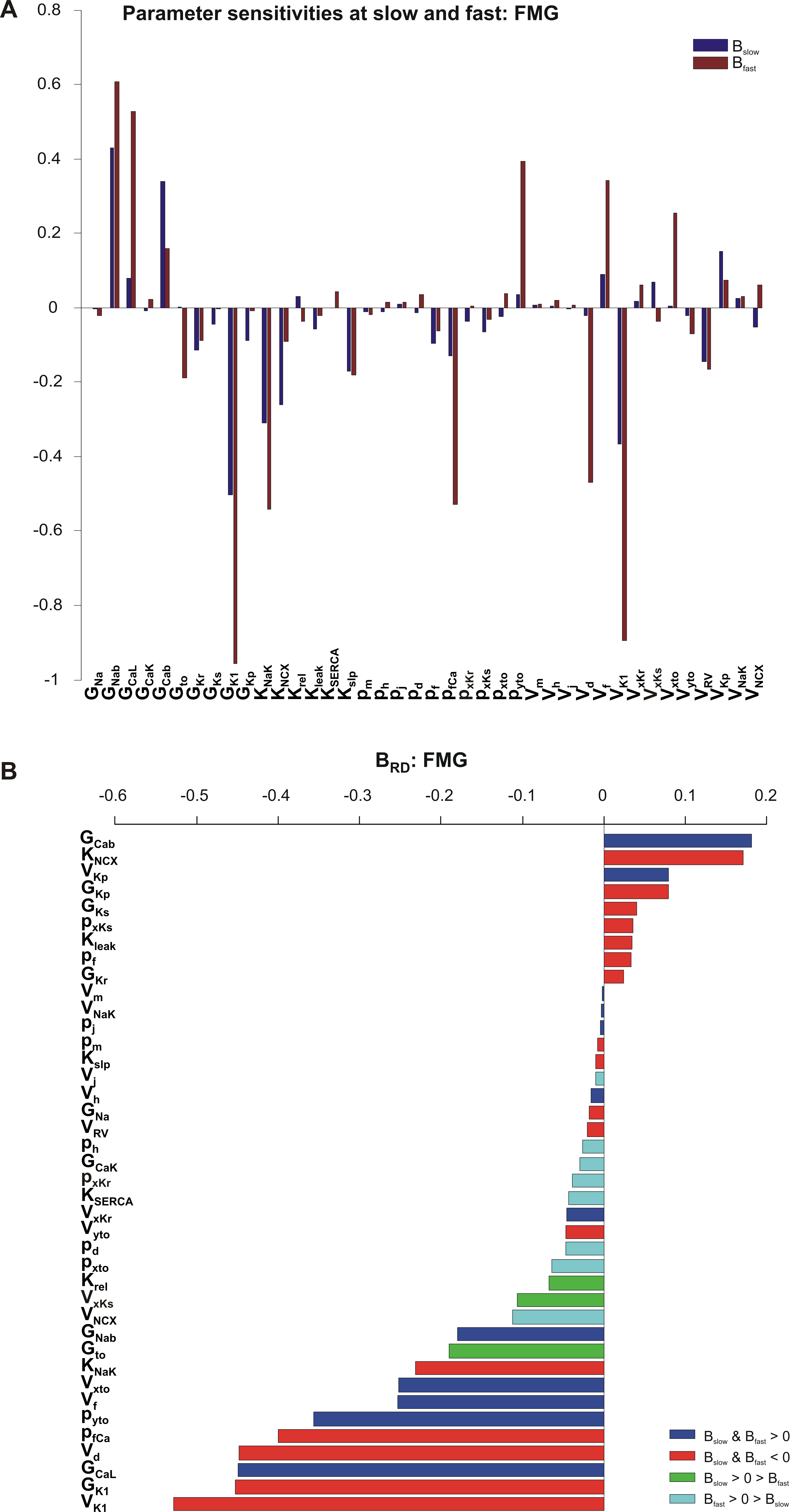


**Figure S13.** **(A)** Parameter sensitivity values and **(B)** rate dependence in the FMG model.
